# ATXN1 N-terminal region explains the binding differences of wild-type and expanded forms

**DOI:** 10.1186/s12920-019-0594-4

**Published:** 2019-10-26

**Authors:** Sara Rocha, Jorge Vieira, Noé Vázquez, Hugo López-Fernández, Florentino Fdez-Riverola, Miguel Reboiro-Jato, André D. Sousa, Cristina P. Vieira

**Affiliations:** 1 Instituto de Biologia Molecular e Celular (IBMC), Rua Alfredo Allen, 208, 4200-135 Porto, Portugal; 2 Instituto de Investigação e Inovação em Saúde (I3S), Universidade do Porto, Rua Alfredo Allen, 208, 4200-135 Porto, Portugal; 3 ESEI - Escuela Superior de Ingeniería Informática, Edificio Politécnico, Campus Universitario As Lagoas s/n, Universidad de Vigo, 32004 Ourense, Spain; 4 Centro de Investigaciones Biomédicas (Centro Singular de Investigación de Galicia), Vigo, Spain; 5 SING Research Group, Galicia Sur Health Research Institute (IIS Galicia Sur). SERGAS-UVIGO, Vigo, Spain

**Keywords:** Protein-protein interaction, Wild-type ATXN1, Expanded ATXN1, Binding interface

## Abstract

**Background:**

Wild-type (wt) polyglutamine (polyQ) regions are implicated in stabilization of protein-protein interactions (PPI). Pathological polyQ expansion, such as that in human Ataxin-1 (ATXN1), that causes spinocerebellar ataxia type 1 (SCA1), results in abnormal PPI. For ATXN1 a larger number of interactors has been reported for the expanded (82Q) than the wt (29Q) protein.

**Methods:**

To understand how the expanded polyQ affects PPI, protein structures were predicted for wt and expanded ATXN1, as well as, for 71 ATXN1 interactors. Then, the binding surfaces of wt and expanded ATXN1 with the reported interactors were inferred.

**Results:**

Our data supports that the polyQ expansion alters the ATXN1 conformation and that it enhances the strength of interaction with ATXN1 partners. For both ATXN1 variants, the number of residues at the predicted binding interface are greater after the polyQ, mainly due to the AXH domain. Moreover, the difference in the interaction strength of the ATXN1 variants was due to an increase in the number of interactions at the N-terminal region, before the polyQ, for the expanded form.

**Conclusions:**

There are three regions at the AXH domain that are essential for ATXN1 PPI. The N-terminal region is responsible for the strength of the PPI with the ATXN1 variants. How the predicted motifs in this region affect PPI is discussed, in the context of ATXN1 post-transcriptional modifications.

## Background

There are nine polyglutamine (polyQ) diseases caused by expansion of a trinucleotide CAG repeat giving rise to a protein with a repeated polyQ tract that extends over its physiological length [1–4]. Although the proteins that cause these disorders are unrelated, polyQ disorders share similarities such as mutational mechanism, progressive neurodegeneration in specific neuronal populations, formation of protein aggregates, negative correlation between length of CAG repeats and age of onset, and positive correlation with the disease severity [5]. One of these, spinocerebellar ataxia type 1 (SCA1), is caused by a polyQ expansion in the human Ataxin-1 (ATXN1) protein.

Wt ATXN1 protein is 816 amino acids long, but its length varies depending on the number of glutamine repeats. In the normal population, the polyQ tract varies from four to 36 glutamine repeats and in SCA1 patients, it can contain between 38 to 83 uninterrupted glutamines [6, 7]. The polyQ tract (residues 197–225) is located at the N-terminal region. Based on sequence conservation between species and secondary structure prediction, the globular AXH domain (residues 562–693) has been identified [8]. This domain is involved in interactions of the ATXN1 with itself, as well as the majority of the identified ATXN1 interactors [9–13]. The self-association region (SAR, residues 494–604, [14]), the RNA-binding region (residues 540–766, [15]), and the C-terminal region (residues 690–816, [16–18] have been also described as important in ATXN1 self-interaction and aggregation of the mutant ATXN1 [19], a hallmark of polyQ diseases. Furthermore, the nuclear localization signal (NLS, residues 794–797, [20]) is the major determinant of ATXN1 transport to the nucleus.

ATXN1 is involved in the formation and regulation of multimeric protein complexes within the nucleus [21]. Abnormal interactions of the mutant ATXN1, is also a hallmark of the SCA1 disease [19]. Two independent large protein complexes have been reported, one with Capicua (CIC), a transcription factor, and another with RBM17 (a splicing factor). ATXN1 interaction with CIC is mediated by the AXH domain [11], and is involved in transcriptional repression and regulation of Notch- and CIC-controlled developmental processes including the nervous system development [22–27]. Aberrant Notch signaling and CIC repressor activity contributes to the pathogenesis of SCA1 [11, 25, 28]. mRNA profiling in mouse models has been used to address the ATXN1-CIC complex, in native and expanded states, and only the complex with expanded ATXN1 is critical for cerebellar SCA1 phenotypes [28]. The interaction between ATXN1 and RBM17 is dependent on the phosphorylation site S776 [29]. Overexpression of RBM17 augments the toxicity of the expanded ATXN1 in *Drosophila* [29]. Other proteins such as those of the 14–3-3 family, that bind to proteins containing phospho-serine motifs, can also interact with ATXN1, and contribute to its stabilization, probably by protecting phosphorylation at site S776. Therefore, protein phosphorylation plays a major role in the function and activity of ATXN1. Other processes such as ubiquitination, sumoylation and transglutamination are also important for the ATXN1 activity [30]. ATXN1 presents typical eukaryotic linear motifs for all these processes [31]. Functional evidence for the role of ubiquitination in SCA1 aggregation came from the treatment with proteasome inhibitors of transfected cells with expanded polyQ ATXN1, that promotes the formation of ATXN1 aggregates. Several enzymes of the ubiquitination system, such as UBCH6, CHIP, and UBE3A have been implicated in the modulation of the ATXN1 transcriptional activity, but the ATXN1 regions involved in such regulation are unknown. In sumoylation, a similar enzymatic cascade to ubiquitination is used, and SUMO-1 (small ubiquitin-like modifier) has been detected in affected brain regions in ATXN1 patients. Although 17 ATXN1 consensus sumoylation sites have been identified, only four (K^16^, K^194^, K^610^, K^697^, and K^746^) have been implicated in decreased sumoylation of ATXN1 [30].

Detection of PPI are based on in-vivo or in-vitro experiments-based methods, as well as *in-silico* methods (see Table 1 in [32] for a summary of the methods), and all have advantages and disadvantages (for a detailed discussion on each method see [33]). PPI for different species, using different detection approaches are publicly available in many databases (e.g BioGRID, CCSB, DroID, FlyBase, HIPPIE, HitPredict, HomoMINT, Instruct, Interactome3D, mentha, MINT, PINA). Most of the databases use different building approaches and although there is overlap between them, each presents a unique set of information [34]. Web platforms such as EvoPPI can be used to obtain all available PPI for a particular protein in a species and/or between species by performing a Blast search [34, 35]. For the human ATXN1, 311 PPI are obtained with EvoPPI when the different publicly available PPI databases are used (Additional file 1: Table S1 [35];). 165 ATXN1 PPI have been identified by performing yeast two-hybrid (Y2H) screens for proteins involved in inherited ataxias, and by validating the results using randomly sampled interacting pairs using co-affinity purification co-AP glutathione-S-transferase (GST) pull-down assays in human HEK293T cells [36]. Also, using human HEK293T cells and quantitative affinity purification and mass spectrometry, Hosp et al. [37] identified 54 proteins that interact with ATXN1, that were not reported by Lim et al. [36], and not included in the main EvoPPI databases. These authors observed that approximately 80% of the interaction partners were shared between the wt and the expanded ATXN1, suggesting that polyQ expansion does not dramatically change the interaction partners of ATXN1. Nevertheless, Suter et al. [38] using ATXN1Q32 and ATXN1Q79 Y2H interaction screens and DNA microarrays for high-throughput quantitative PPI detection, reported only seven out of 81 proteins in common in the two experiments. Changes in PPI are observed in SCA1 [39–41], and thus, comparison of PPI networks in controls and patients have been used to get insights into the basis of this disease [36, 38]. In these studies, the ATXN1 interactors bind with higher affinity the expanded than the wt form. Nevertheless, Hosp et al. [37] were not able to validate this finding, raising the issue of whether the previous results can be explained on the basis of different binding affinities. The human paralog of ATXN1, Ataxin-1-like (ATXN1L), does not encode a protein with a polyQ tract, shares with ATXN1 conserved domains (the NBA at the N-terminal region, the SAR and AXH), several interactors, and their expression profiles are very similar [42]. Indeed, ATXN1 and ATXN1L seem to be functionally redundant, since, in flies and mice, increased ATXN1L levels induce the sequestration of expanded ATXN1 into nuclear inclusions, possibly by replacing ATXN1 from the endogenous complexes containing CIC [43].

**Table 1:** PPI with the wt and expanded ATXN1 according to the methodologies used

ATXN1 binding preference	Proteins names (GeneID; UniProtKB)
wt (in both approaches)	CRY2 (1408; Q49AN0); EIF1B (10289; O60739); GGA2 (23062; Q9UJY4); LITAF (9516; Q99732); RBM26 (64062; Q5T8P6); SEMA4G (57715; Q9NTN9); TOMM20 (9804; Q15388)
Expanded (in both approaches)	ADD3 (120; Q9UEY8); ARID5A (10865; Q03989); ASNS (440; P08243); BAALC (79870; Q8WXS3); BASP1 (10409; P80723); C16orf5 (29965; Q9H305); C2orf27B (408029; Q580R0); CAMK2B (816; Q13554); CHRNA7 (1139; P36544); CRK (1398; P46108); DHRSX (207063; Q8N5I4); DHX37 (57647; Q8IY37); DIXDC1 (85458; Q155Q3); EIF3F (8665; O00303); ESRRA (2101; P11474); ETV4 (2118; P43268); FAM46B (115572; Q96A09); FAR1 (84188; Q8WVX9); FOSL1 (8061; P15407); GATAD1 (57798; Q8WUU5); HEY2 (23493; Q9UBP5); HEYL (26508; Q9NQ87); HNRPLL (92906; Q8WVV9); ILVBL (10994; A1L0T0); IMMT (10989; Q16891); KCTD15 (79047; Q96SI1); KIF22 (3835; Q14807); LASP1 (3927; Q14847); LPAR2 (9170; Q9HBW0); MAGEB18 (286514; Q96M61); MAGEB2 (4113; O15479); MAGEB6 (158809; Q8N7X4); MCART1 (92014; Q9H1U9); MLST8 (64223; Q9BVC4); NCAM1 (4684; P13591); OTX2 (5015; P32243); PIAS1 (8554; O75925); PPAT (5471; Q06203); QKI (9444; Q96PU8); RAPGEF1 (2889; Q13905); RBFOX2 (23543; O43251); SF1 (7536; Q15637); SLC6A13 (6540; Q9NSD5); STAM2 (10254; O75886); TMX2 (51075; Q9Y320); TRIM38 (10475; O00635); TSC1 (7248; Q92574); TTRAP (51567; O95551); UHRF2 (115426; Q96PU4); WBSCR16 (81554; Q96I51); YWHAE (7531; P62258); ZC3H10 (84872; Q96K80); ZSCAN1 (284312; Q8NBB4)
wt in 1/ Expanded in 2	CREM (1390; Q03060); CRIP2(1397; P52943); MSX2 (4488; P35548); PLEKHB1 (58473; Q9UF11); PSPH (5723; P78330); SV2A (9900; Q7L0J3)
wt in 2/ Expanded in 1	CXorf27 (25763; O75409); RAI2 (10742; Q9Y5P3); TCTA (6988; P57738); TP53I11 (9537; O14683)
wt in 1/ Expanded in 1 and 2	FAM46A (55603; Q96IP4)

1) the most reliable structure according to HADDOCK, 2) docking structure that maximizes the probability of having a PPI

Here we use an *in-silico* approach to address whether the expanded ATXN1 protein is predicted to bind with higher affinity to reported interactors. Moreover, these predictions can shed light on the ATXN1 regions that could be responsible for the higher binding affinity. We compare our results primarily with those of Suter et al. [38], since these authors report the largest set of interactors using full-length proteins and not truncated protein segments/domains as baits. Our results support the observation made by Suter et al. [38] and Lim et al. [36] that the expanded ATXN1 shows larger number of interactors than the wt form, that the number of interface residues is larger in the expanded ATXN1, as well as the importance of the AXH domain for protein binding. We suggest that the N-terminal region of ATXN1 is the one responsible for the binding differences of the wt and expanded ATXN1. Based on the amino acid motifs at this region, as well as the inferred RNA binding region, we discuss how interactions with expanded polyQ region can affect ATXN1 function.

## Methods

### ATXN1 PPI

*H. sapiens*, *M. musculus*, *X. laevis*, *D. rerio, D. melanogaster,* and *C. elegans* ATXN1 and ATXN1L PPI have been retrieved from EvoPPI ([34, 35]; http://evoppi.i3s.up.pt); accession numbers are listed in Additional file 10: Table S5). For the between species comparison of the interactomes of ATXN1 and ATXN1L we also used EvoPPI, with the following parameters: 0.05 for the minimum expect value (evalue); 50 for the minimum length of aligment block; 40 for the minimum identity (%) and 1 for the number of descriptions (max_target_seqs) and the interaction level. Venny web tool (v.2.1.0: http://bioinfogp.cnb.csic.es/tools/venny/) was used to identify the common interactors.

### *In-silico* approaches to predict the wt and expanded ATXN1 interacting surfaces with 71 interacting partners

The 81 proteins reported in Suter et al. [38] were obtained from UniProtKB. We used ATXN1Q29 as wt and ATXN1Q82 as expanded ATXN1. The 3D structure prediction of these proteins was obtained with I-TASSER [44], except for DOCK5 and IGF2R that are longer than 1500 residues (the limit size for I-TASSER analyses). The models with higher confidence (C-score value; calculated based on the significance of threading template alignments and the convergence parameters of the structure assembly simulations used to estimate the quality of predicted models by I-TASSER. C-score typically ranges from − 5 to 2, where a higher value signifies a model with a high confidence) were used. To evaluate the structure similarity of wt ATXN1 and expanded ATXN1 we used TM-align on-line [45]. The structural images were obtained using the PyMOL molecular package (The PyMOL Molecular Graphics System, Version 1.7.4 Schrödinger, LLC.).

For the docking prediction of wt ATXN1 and expanded ATXN1 with the interacting partners, we used HADDOCK [46]. Putative active (directly involved in the interaction) and passive (surrounding surface residues) residues declared in HADDOCK were obtained with CPORT [47]. For NPHP3 and UHRF1BPL1L we failed to predict the active and the passive residues, and thus, these two proteins, were not further analyzed.

For CLCN2, EHMT1, FUBP3, LOXL1, SLC4A2, and ZXDC we failed to predict the docking with ATXN1Q29 and/or ATXN1Q82, and thus we ended up with results for 71 ATXN1 interactors. For wt and expanded ATXN1, HADDOCK docking structures were selected based on 1) most reliable structure according to HADDOCK (the structure with the lowest Z-score, that indicates how many standard deviations from the average the cluster is located in terms of score), and 2) the structure presenting the highest probability of having a protein-protein interaction between wt ATXN1 and expanded ATXN1. The number and percentage of interface residues (including those that make hydrogen bonds, and salt bridges), the solvent-accessible area (Å), and the percentage of the solvent-accessible area of the wt ATXN1 and the expanded ATXN1 with the interacting protein partners were determined using PISA [48]. If these values are greater for the Docking with the expanded ATXN1 than for the wt ATXN1 we consider that there is a higher binding affinity with the former, and vice versa. We assume no preference if the value is identical for the wt ATXN1 and expanded ATXN1. A flowchart showing methodology 2 (the one showing the best agreement with Suter et al. [38]; see Results) is presented as Additional file 11: Figure S6. The PDB files describing the docking of these proteins are available as Additional file 12. Dataset S1.

The interface residues of the 19 model structures of the AXH domain in complex with CIC (PDB ID: 2 M41, [17], PDB ID: 4J2L, [49]) were also determined using PISA [48].

### Statistical analyses

To address whether the presence of a polyQ influences the observed (according to EvoPPI) number of interactor proteins, regression analyses of the number of ATXN1 interactors in humans and mouse were performed for 65 human proteins that have a polyQ, divided into two groups according to whether in mouse they also have a polyQ. 1000 random permutations were used to compare the slopes and the intercept of the regressions in humans and mouse. Regression analyses were also performed to address whether in humans, the number of interactors is influenced by protein size.

In order to address possible technical biases, non-parametric Mann-Whitney U tests were performed to look for possible associations between sequence length, the I-TASSER C-score, the HADDOCK Z-score, and being in agreement or not with the results of Suter et al. [38].

To test whether the docking predictions that are in agreement with Suter et al. [38] are enriched in coiled-coil domains when compared to the results that are not in agreement with Suter et al., Marcoil1.0 [50] was used to predict the presence of coiled-coil regions in the protein partners of wt and expanded ATXN1. As parameters, we used the 9FAM as Coiled-Coil Emission P. Matrix and the precomputed (MARCOIL-H) as HMM Transition P. Matrix. We only considered as proteins that form coiled-coils regions, those that present coiled-coil predicted domains with a minimum threshold of 90%. A Fisher exact test was used to compare the frequency of cases in the two groups. Moreover, in order to test whether the docking predictions that are in agreement/disagreement with Suter et al. [38] are enriched in a particular gene ontology category, we used PANTHER [51], http://www.pantherdb.org/.

To infer binding preference between wt and expanded ATXN1 forms and the interactors, non-parametric Sign tests were also used to compare the number of residues in the interface of the interacting partners, percentage of number of interface residues, solvent-accessible area (Å), and percentage of the solvent-accessible area.

In order to identify the regions responsible for the higher binding affinity we also compared, using non-parametric sign tests the number of interface residues before and after the polyQ tracts, and the polyQ tracts and 20 amino acids residues flanking the polyQ tract for the wt and expanded ATXN1 forms.

All analyses were performed using SPSS software (https://www.ibm.com/analytics/spss-statistics-software).

### RNA binding predictions

RNA binding protein predictions were performed using RNApred (http://crdd.osdd.net/raghava/rnapred/), using a sliding window of 50 amino acids and an increment of 25.

## Results

### Human polyQ proteins have more interactors than non-polyQ proteins

**Fig. 1:**
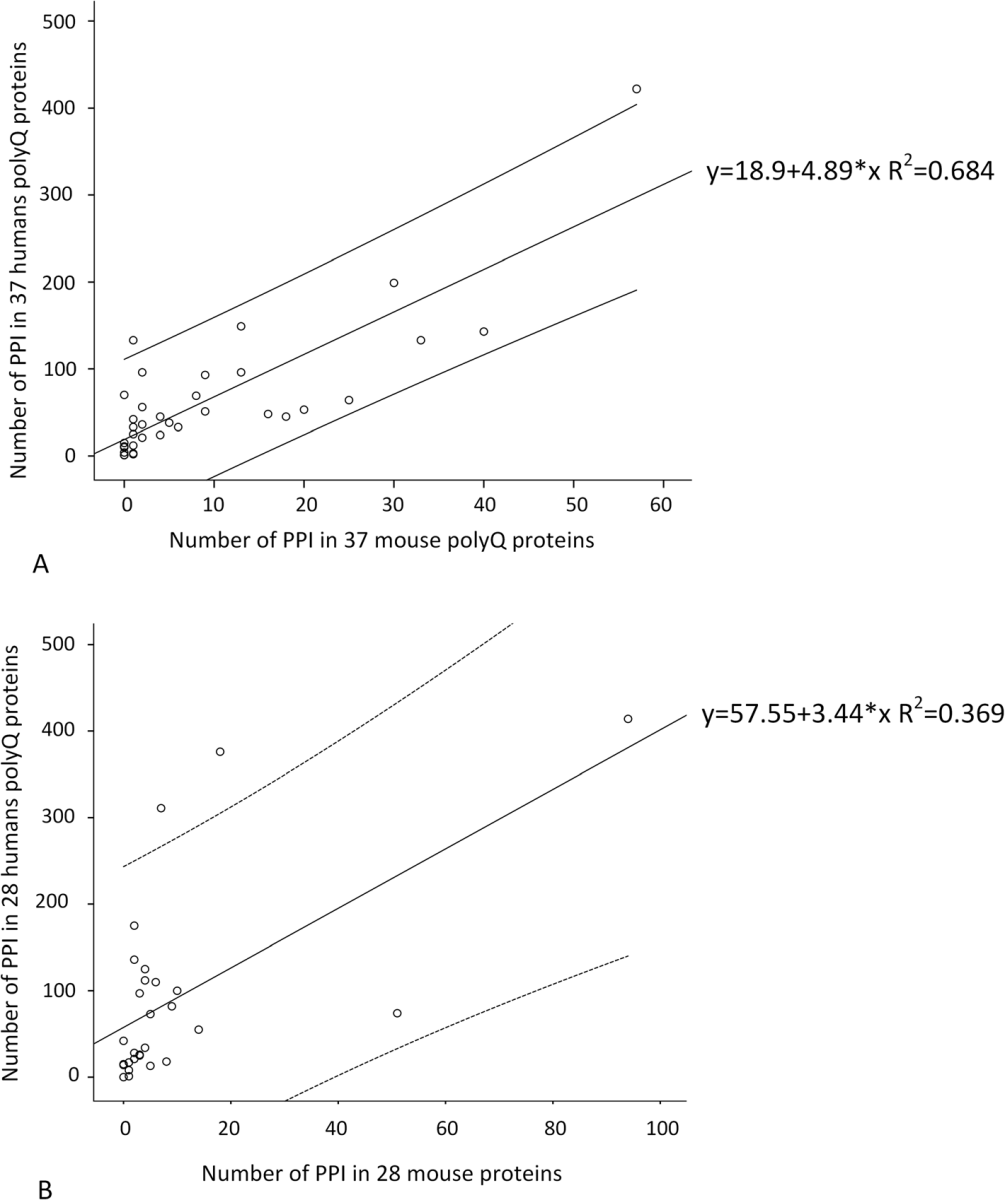
Linear regression equation and 95% confidence interval for the *Homo sapiens* and *Mus musculus* PPI **a** 37 orthologue proteins with a polyQ tract in both species. **b** the 28 proteins that have polyQ in *H. sapiens* but not in *M. musculus*

In humans, polyQ proteins tend to have more interactors than proteins lacking a polyQ tract [35, 40] which supports the view that the presence of a polyQ region increases the number of partner proteins. Nevertheless, in humans, more than 73% of the 65 genes encoding polyQ proteins are associated with diseases (Additional file 2: Table S2), and thus it is unlikely that the reported interactions are a random sample of the interactome. Therefore, we retrieved from EvoPPI ([34, 35]; http://evoppi.i3s.up.pt), the number of interactors for the 65 human proteins that have a polyQ (a stretch with a minimum of 10 consecutive glutamines) as well as for the proteins encoded by the *Mus musculus* orthologs. Then, we compare the number of interactors for the proteins presenting a polyQ in both species, and those presenting a polyQ in humans only (Fig. 1a, b, respectively). For proteins having polyQ in both humans and mouse (37), the regression of the number of interactions in the two species explains 68.4% of the variability, while for proteins that have polyQ in humans but not in mouse explains 36.9% of the variability. The slope of the former (4.89) is higher than for the latter (3.44). Nevertheless, when we pull all data together and get 1000 random samples of size 37, a slope as high or higher than 4.89 is obtained 11.4% of the times. Moreover, when we obtain 1000 random samples of size 28, a slope as low or lower than 3.44 is obtained 27.0% of the times. Therefore, the observed slopes for the regression of the number of interactions of proteins having polyQ in both humans and mouse and proteins that have polyQ in humans but not in mouse, could have been generated by taking two random samples of size 37 and 28 from a single population, meaning that the two regression lines are likely parallel. The slope of these regressions indicate the difference in the amount of data available for humans and mouse (in between 3.44 and 4.89 more data for humans than for mouse). The interception of the y axis is much lower (18.9) for the regression obtained when using proteins having polyQ in both humans and mouse than when using proteins that have polyQ in humans but not in mouse (57.55). When we pull all data together and get 1000 random samples of size 37, an interception as low or lower than 18.9 is obtained in only 0.1% of the cases. Moreover, when we obtain 1000 random samples of size 28 an interception as high or higher than 57.55 is obtained only 2.9% of the times. It is thus highly unlikely that the two datasets represent two random samples. Therefore, proteins that have polyQ in humans but not in mouse have many more interactions in humans than what is expected considering the number of reported interactions in mouse and the regression obtained for proteins having polyQ in both humans and mouse. In humans, the number of interactors is not related to the size of the proteins (size according to UniProtKB; linear regression analyzes *P* = 0.084; Additional file 3: Figure S1), in agreement with Schaefer et al. [40].

The interactomes of the *Homo sapiens* ATXN1 and ATXN1L retrieved from EvoPPI [34], revealed a minimum of 311 and 70 interactors, respectively. Despite the conservation between these proteins [42] only 35 interact with both ATXN1 and ATXN1L (Additional file 4: Table S3). Seven of these proteins (RBFOX2, SREBF1, NCOR1, GMEB2, PHPT1, ANKHD1, and STAC2) are reported as interacting with expanded ATXN1 [36, 38], despite ATXN1L not having a polyQ tract. Moreover, of the different *M. musculus* atxn1 interactors (YWHAE, RBM17, CIC, VCP, KAT5, and Nr1f1) YWHAE and RBM17 are reported as interacting in humans with expanded ATXN1 [38], although the mouse atxn1 does not have a polyQ tract. Moreover, none of these interactors are predicted to have coiled-coil regions, that have been implicated in the interaction with polyQ regions [40, 52, 53]. For the three *Drosophila melanogaster* interactions reported for the orthologous ATXN1, none is in common with the human ATXN1. For *Xenopus tropicalis*, *Danio rerio*, *and Caenorhabditis elegans* no information is available for the orthologous ATXN1, as well as for the orthologous ATXN1L for *M. musculus*, *Xenopus laevis*, *D. rerio*, and *C. elegans*.

### Validation of the *in-silico* method used for predicting protein binding surfaces

Although two methodologies were used for the identification of the protein interfaces, namely: 1) the most reliable complex structure according to HADDOCK, and 2) the complex structure that maximizes the probability of having a PPI, for 73.2% (60 out 71) of the proteins reported by Suter et al. [38] as interacting with wt ATXN1(Q29) (7; Table 1) and/or expanded ATXN1(Q82) (53; Table 1) the results were the same (Table 1). For the remaining cases, to address the accuracy of the two approaches, the predictions generated by them regarding binding strength towards the wt and expanded ATXN1, are compared with the wet bench results of Suter et al. [38]. When we used approach 1), 38 out of the 71 PPI analyzed were in agreement in both studies (53.5%) and when we use approach 2) 43 out of the 71 PPI analyzed were in agreement in both studies (60.6%) (Table 2). Therefore, the second approach is more reliable in predicting the interaction of proteins with wt ATXN1 (three of the 16 proteins (18.7%)) and expanded ATXN1 (40 of the 48 (83.3%)).

**Table 2:** Predicted (approach 2) and determined (Suter et al. [38]) binding preferences of ATXN1 partners with the wt and expanded ATXN1. In bold are those proteins for which the prediction is different when using the two *in-silico* methodologies

This work	Suter et al.	Proteins names (GeneID; UniProtKB)
wt	wt	**CXorf27 (25763; O75409)**; GGA2 (23062; Q9UJY4); TOMM20 (9804; Q15388)
expanded	expanded	BAALC (79870; Q8WXS3); BASP1 (10409; P80723); C16orf5 (29965; Q9H305); C2orf27B (408029; Q580R0); **CREM (1390; Q03060)**; **CRIP2 (1397; P52943)**; CRK (1398; P46108); DHRSX (207063; Q8N5I4); ESRRA (2101; P11474); **FAM46A (55603; Q96IP4)**; FAM46B (115572; Q96A09); FAR1 (84188; Q8WVX9); GATAD1 (57798; Q8WUU5); HEY2 (23493; Q9UBP5); HNRPLL (92906; Q8WVV9); IMMT (10989; Q16891); KCTD15 (79047; Q96SI1); KIF22 (3835; Q14807); LPAR2 (9170; Q9HBW0); MCART1 (92014; Q9H1U9); **MSX2 (4488 (P35548)**; NCAM1 (4684; P13591); OTX2 (5015; P32243); PIAS1 (8554; O75925); **PLEKHB1 (58473; Q9UF11)**; PPAT (5471; Q06203); **PSPH (5723; P78330)**; QKI (9444; Q96PU8); RAPGEF1 (2889; Q13905); RBM9 (23543; O43251); SF1 (7536; Q15637); SLC6A13 (6540; Q9NSD5); **SV2A (9900; Q7L0J3)**; TMX2 (51075; Q9Y320); TRIM38 (10475; O00635); TTRAP (51567; O95551); UHRF2 (115426; Q96PU4); YWHAE (7531 (P62258); ZC3H10 (84872; Q96K80); ZSCAN1 (284312; Q8NBB4)
wt	expanded	CRY2 (1408; Q49AN0); EIF1B (10289; O60739); LITAF (9516; Q99732); **RAI2 (10742; Q9Y5P3**); RBM26 (64062; Q5T8P6); SEMA4G (57715; Q9NTN9); **TCTA (6988; P57738)**; **TP53I11 (9537; O14683)**
expanded	wt	ADD3 (120; Q9UEY8); ASNS (440; P08243); CAMK2B (816; Q13554); DHX37 (57647; Q8IY37); DIXDC1 (85458; Q155Q3); EIF3F (8665; O00303); ETV4 (2118; P43268); HEYL (26508; Q9NQ87); ILVBL (10994; A1L0T0); MAGEB18 (286514; Q96M61); MAGEB2 (4113; O15479); MAGEB6 (158809; Q8N7X4); WBSCR16 (81554; Q96I51)
expanded	no preference	ARID5A (10865; Q03989); CHRNA7 (1139; P36544); FOSL1 (8061; P15407); LASP1 (3927; Q14847); MLST8 (64223; Q9BVC4); STAM2 (10254; O75886); TSC1 (7248; Q92574)

**Fig. 2:**
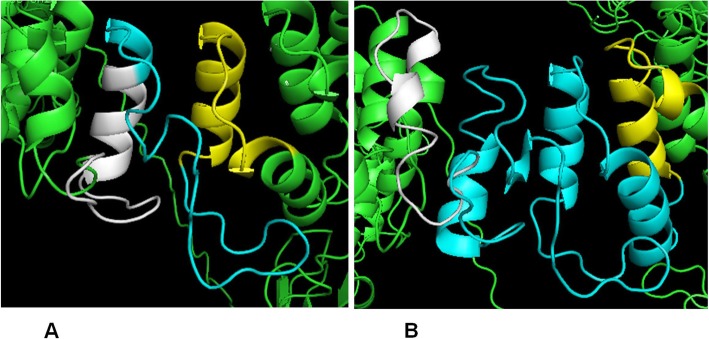
The 3D structure of the polyQ tract and flanking residues. Representation of the 3D structure of the polyQ tract (in cyan) and 20 amino acids residues flanking the polyQ tract (in yellow those in the N-terminal region, and in white those in the C-terminal region) in: **a** wt ATXN1 and **b** expanded ATXN1

Moreover, the use of the same methodology but different parameters such as, the number of residues in the interface of the interacting partners, percentage of number of interface residues, solvent-accessible area (Å), and percentage of the solvent-accessible area of wt ATXN1, expanded ATXN1 and interacting partners, does not result in a better agreement with Suter et al. 2013 results (Additional file 5: Table S4). The sequence length of the interacting proteins (*P* = 0.312), the I-TASSER C-score of the interacting proteins (that estimates the quality of predicted models by I-TASSER, calculated based on the significance of threading template alignments and the convergence parameters of the structure assembly simulations; *P* = 0.892), and the Haddock Z-score (that indicates how many standard deviations from the average the cluster is located in terms of score) of both forms of ATXN1 (wt, *P* = 0.911; expanded, *P* = 0.608) are not statistically significantly associated with the ability of making the correct prediction (Mann-Whitney U test). The main difference in the I-TASSER predicted structures of the wt ATXN1 (Q29) and expanded ATXN1 (Q82) presenting the most similar structures (TM-score = 0.97361; if normalized by length of wt ATXN1 [45]; and the highest C-score), is that the wt polyQ tract is predicted as random coil (absence of regular secondary structures), while in the expanded polyQ this region is predicted as three α-helices connected by loops (Fig. 2). It should be noted that I-TASSER modeling of the 3D structures starts from the structure templates from the PDB library and uses the templates of the highest significance in the threading alignments [44], and the polyQ region has fewer amino acids used as threading templates (Additional file 6: Figure S2), making the prediction of these regions less reliable. Therefore, the *in-silico* methodology is likely more robust when no interactions are predicted with the polyQ tract.

Since alpha-helical coiled-coil domains have been suggested as being critical for the spontaneous aggregation of Q/N-rich yeast prions and polyQ disease proteins [40, 52, 53] we have addressed if the proteins that show similar results in the *in-silico* and Suter et al. [38] approaches are enriched in such domains. Nevertheless, using a Fisher exact test, the presence of predicted coiled-coil domains (using Marcoils prediction threshold of 90%) is not statistically different (*P* = 0.347) in the two datasets.

We also address if when our results are/are not in agreement with those of Suter et al. [38], the interacting proteins are enriched in a particular function. According to PANTHER [51] there is no statistically significant enrichment for the two datasets (for all gene ontology classes *P* > 0.05).

### The AXH domain is important for PPI

**Fig. 3:**
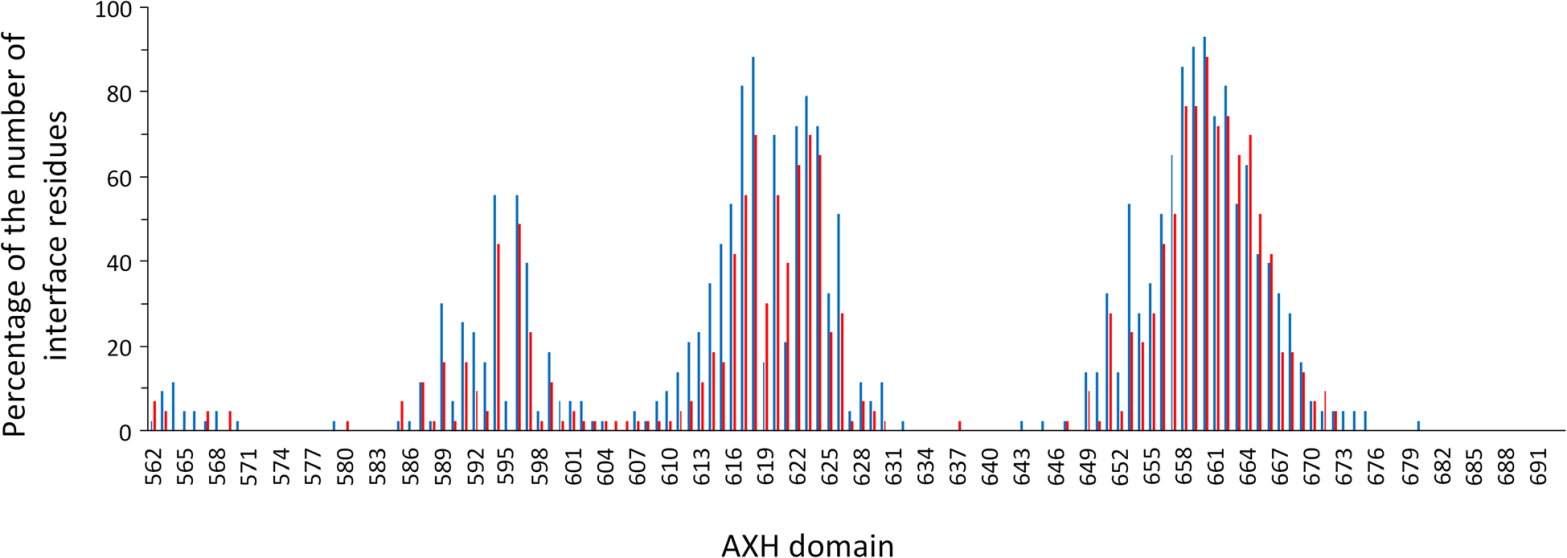
AXH domain interface residues of the 43 interactors in agreement with Suter et al. [38]. The percentage of the number of interface residues at the AXH domain with the 43 interactors in agreement with Suter et al. [38] for the wt (in blue) and expanded (in red) ATXN1

The AXH domain region, relevant to PPI, presents a similar pattern of interaction for both wt and expanded ATXN1 types. In Lim et al. [36], 72.5% of the ATXN1 protein partners bind the AXH domain. It should be noted that the AXH domain can mediate neurodegeneration through its interaction with other proteins [54, 55]. Here we show that most of the proteins studied interact with the AXH domain at three regions (Fig. 3). An overlap is observed at seven residues (ATXN1 sites 588, 591, 594, 599, 602, 609, and 649; see Additional file 7: Figure S3) between our predictions and those identified when using the 19 available crystal structures for the complex ATXN1/AXH domain and CIC [17, 49]. Therefore, these regions are fundamental for the PPI of ATXN1.

### The expanded ATXN1 shows more residues at the binding interface than the wt form

**Fig. 4:**
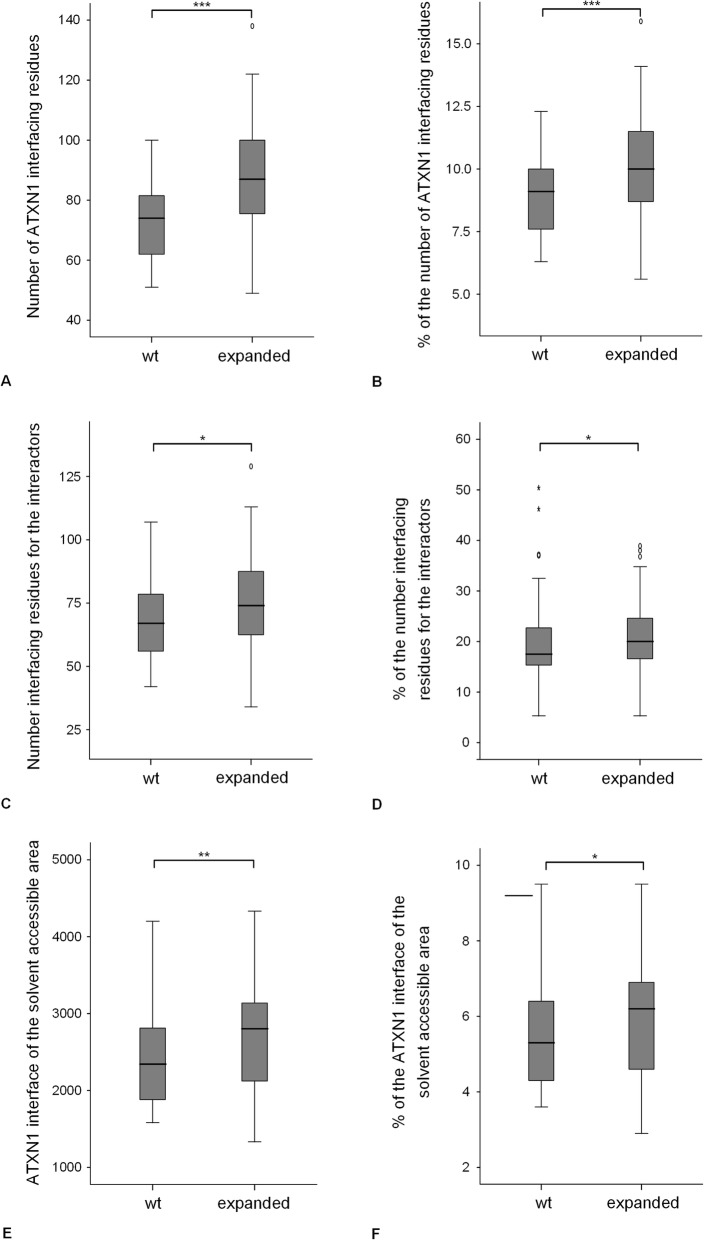
Comparison of the wt and expanded ATXN1 docking results of the 43 interactors analyzed **a** interfacing residues of the ATXN1. **b** percentage of interfacing residues of the ATXN1. **c** number of interfacing residues for the 43 interactors. **d** percentage of interfacing residues of the 43 interactors. **e** interface of the solvent accessible area (Å) of the ATXN1. **f** percentage of the interface of the solvent accessible area of the ATXN1

Comparison of the docking results of the expanded and wt ATXN1 with the 43 proteins for which the *in-silico* results are in agreement with those of Suter et al. [38], revealed a significant increase in the interfacing residues and in the percentage of interfacing residues of the expanded ATXN1 compared with wt ATXN1 (Sign test; *P* = 0.000 for both; Fig. 4a, b). Moreover, the number and the percentage of interfacing residues of the 43 interacting proteins with the expanded ATXN1 is increased compared with wt ATXN1 (*P* = 0.015 for both; Fig. 4c, d). The buried solvent accessible area (Å) at the predicted binding interface of the expanded ATXN1 (*P* = 0.002; Fig. 4e) also increases compared with the wt ATXN1. Moreover, the percentage of the solvent accessible area buried in the docking complexes of the expanded mutated ATXN1 (*P* = 0.025) increases compared with the wt ATXN1 (Fig. 4f). It should be noted that the analysis of the docking results of the expanded and wt ATXN1 when using all the 71 proteins revealed similar results (Additional file 8: Figure S4A-F) except for the percentage of the buried solvent accessible area of the expanded ATXN1 (*P* = 0.104) that does not increase compared with the wt ATXN1.

### The N-terminal region of the expanded ATXN1 shows more residues at the predicted binding interface and an increase in the number of hydrogen bonds and salt bridges when compared to the wt

**Fig. 5:**
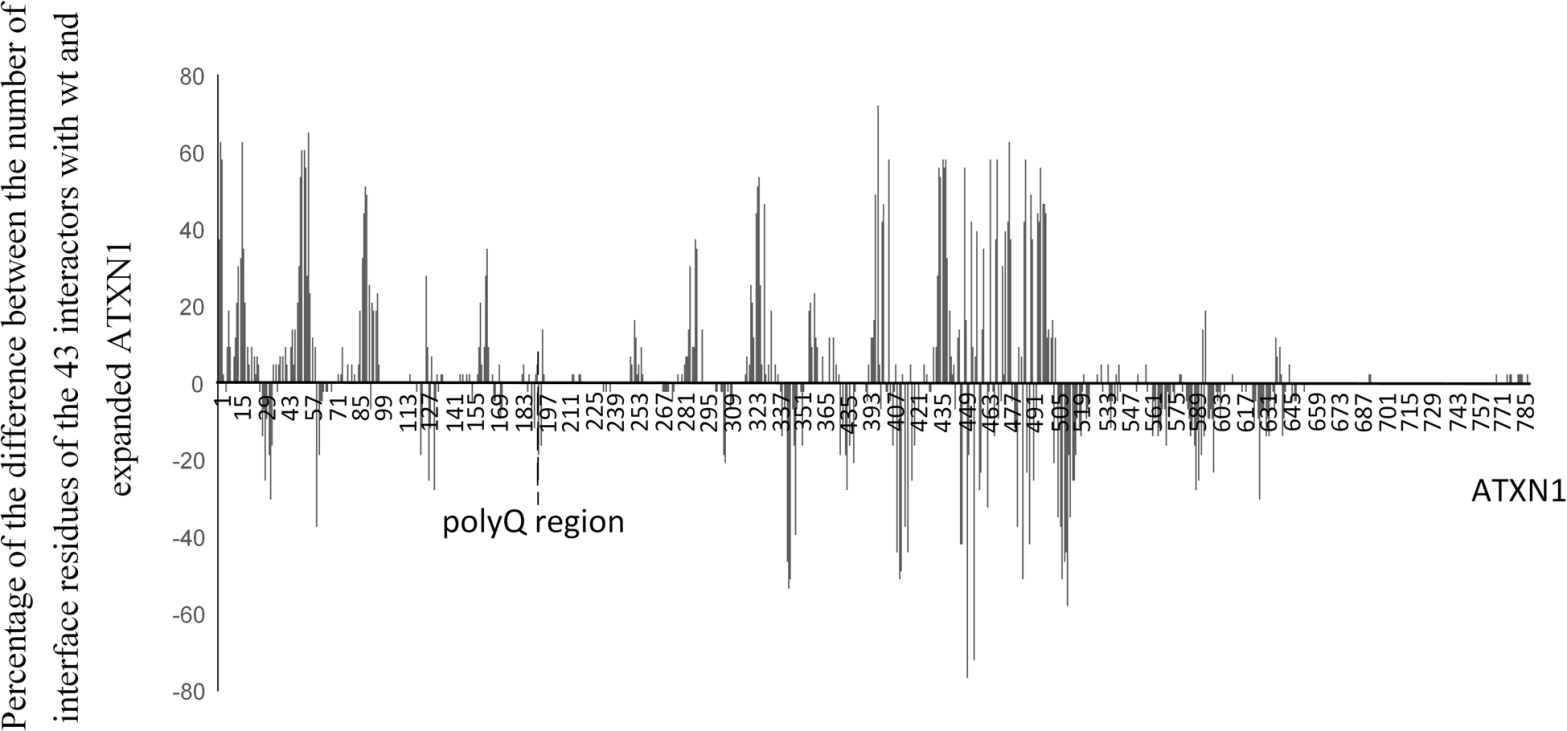
Difference of the number of interface residues of the 43 interactors analyzed. Percentage of the difference of the number of interface residues of the 43 interactors in agreement with Suter et al. [38], with wt and expanded ATXN1. PolyQ tract is not represented

To identify the regions of the ATXN1 that are responsible for the overall significant increase in the number of interfacing residues of the expanded ATXN1 when compared with the wt ATXN1, we analyzed separately the sum of the number of interface residues in six regions: before and after the polyQ tracts (including the 20 amino acids residues flanking the polyQ tract), the polyQ tracts, the 20 amino acid residues before and after the polyQ tracts and the AXH domain (Table 3). Using the docking results of the expanded ATXN1 and wt ATXN1 with the 43 proteins that are in agreement with Suter et al. [38], a significant increase is only observed in the number of residues before the polyQ tract (Non-parametric Sign test; for both *P* = 0.000; Fig. 5).

**Table 3:** Number of interface residue (%) for the PPI in common with Suter et al. [38]

ATXN1	Before the polyQ	PolyQ tract	After the polyQ	20 amino acid before polyQ	20 amino acid after polyQ	AXH domain
wt	12.2	0.2	87.6	0.7	0.0	31.1
Expanded	23.6	1.0	75.3	0.5	0.1	20.2

The percentage of the difference of the number of predicted interface residues of the 43 proteins when they interact with expanded and wt ATXN1 revealed that the largest changes occurred in the N-terminal region of ATXN1 (Fig. 5). Moreover, the number of predicted hydrogen bonds as well as salt bridges is higher at the N-terminal region of the expanded form than in the wt (Non-parametric Sign test; for both *P* = 0.000). This is in agreement with Lim et al. [36], which using an Y2H assay, also describe a predominance of proteins interacting with the N-terminal region of the expanded ATXN1 (55.7%) when compared with wt ATXN1 (15.2%). Therefore, it seems that the presence of a polyQ region affects mostly the interaction in the region before the polyQ tract. In this region, motifs related with transcription, cell communication, phosphorylation, and sumoylation (Table 4) were identified, using the eukaryotic linear motifs (elm.eu.org) prediction tool. Moreover, using RNApred (a webserver for prediction of RNA-binding proteins), a sliding window of 50 residues and an increment of 25, four putative RNA binding regions are inferred, namely one before the polyQ region and three after (Additional file 9: Figure S5). One of them corresponds to the region described in the literature.

**Table 4:** ATXN1 N-terminal region eukaryotic linear motifs (elm.eu.org)

Pathway	Sequence motif	Location	Proteins that bind in the region	Description	Pattern	Probability
Transcription	YSPPSAP	87–93	32	Motif recognized by class I SH3 domains	[RKY]..P..P	1.237e-03
	AWLPGNP	44–50	21		...[PV]..P	1.317e-02
Phosphorylation	SPPSAPR	88–94	31	CK1 recognition site	S..([ST])...	1.704e-02
	SPPSAPRS	88–95	32	GSK3 recognition site	...([ST])...[ST]	2.679e-02
	PVTSAVAS	154–161	21			
Cell communication	PSAP	90–93	27	Glycosaminoglycan attachment site	[ED].(S)[GA]	1.792e-02
	ASAA	160–163	24			
Sumoylation	TFQFI	126–130	21	Glycosaminoglycan attachment site	[ED].(S)[GA]	1.792e-02

ATXN1 N-terminal region eukaryotic linear motifs (elm.eu.org) for which interaction is observed in more than 20 interactors showing strong interaction with expanded ATXN1

## Discussion

There is only a partial agreement between our predictions and the experimental data retrieved from Suter et al. [38]. Therefore, in what follows we discuss the possible sources of error. First, we must consider the wrong prediction of the protein structure for some interactors. Nevertheless, the quality of the prediction is usually evaluated by the C-score value, and we found no association between being or not in agreement with Suter et al. [38] (independently of considering the difference in the number (*P* = 0.892), percentage of interfacing residues (*P* = 0.325), the buried solvent accessible area (*P* = 0.2), or the percentage of the solvent accessible area buried in the docking complexes (P = 0.2) and the C-score value (non-parametric Mann-Whitney tests).

The second possible source of error is the quality of the prediction of the docking structures, evaluated by the associated Z-score. For both the wt and expanded ATXN1, when we use the docking structure presenting the lowest Z-score or the docking structure with the highest probability of having a protein-protein interaction, we found no association between the Z-score and being or not in agreement (independently of considering the difference in the number of interacting residues for ATXN1 (wt, *P* = 0.911; expanded, *P* = 0.608) or for the interactors (wt, *P* = 0.086; expanded, *P* = 0.848), percentage of interfacing residues for ATXN1 (wt, *P* = 0.339; expanded, *P* = 0.784) or for the interactors (wt, *P* = 0.086; expanded, *P* = 0.848), the buried solvent accessible area for ATXN1 (wt, *P* = 0.751; expanded, *P* = 0.708) or for the interactors (wt, *P* = 0.470; expanded, *P* = 0.777), or the percentage of the solvent accessible area buried in the docking complexes for ATXN1 (wt, *P* = 0.372; expanded, *P* = 0.854) or for the interactors (wt, *P* = 0.548; expanded, *P* = 0.894)) with Suter et al. [38] (non-parametric Mann-Whitney tests). Another possible source of error is the wrong inference of the binding surfaces. The prediction that the majority of the proteins bind at the AXH region, suggests that this is not a major source of error either. Nevertheless, the number of interacting residues at the polyQ region is significantly associated with the wrong outcome (Mann-Whitney test; *P* = 0.036), but this association is no longer significant when interactors showing six or more predicted interactions with the ATXN1 polyQ region are removed (P = 0,804). It should also be noted, that ATXN1 likely participates in a multi-protein complex but our predictions are based on the binding of ATXN1 to a single interactor at a time. Although ATXN1 forms dimers mediated by the AXH domain, it has been reported that it binds with other proteins as a monomer [17, 56]. Lastly, the different quantitative methods that have been developed to identify the strength of the binding are not error-free, and thus, for a few cases, the outcome reported in Suter et al. [38] may be wrong. Indeed, the comparison of the results using different methodologies, as for instance with Lim et al. [36], revealed that six of the seven proteins that were detected by Suter et al. [38] as partners of expanded ATXN1 were detected as partners of both forms of ATXN1 by Lim et al. [36] (CRK; FAM46A; FAM46B; OTX2; RBFOX2; ZC3H10) and ARID5A detected by Suter et al. [38] as an interacting partner of both forms of ATXN1, was detected by Lim et al. [36] as interacting with the expanded ATXN1 only. The *in-silico* approach here used has a better performance for proteins that interact preferably with expanded ATXN1 (83.33%) compared with the wt ATXN1 (18.75%). It is unclear whether this is a bias of the *in-silico* method, or whether the number of interactors that interact preferably with the expanded ATXN1 is underestimated by Suter et al. [38]. Our results agree with the hypothesis that the expansion of the polyQ tract that occurs in SCA1 alters the conformation of ATXN1, leading to abnormal strength interactions and conferring a toxic gain of function [10, 18, 29, 57, 58]. It should be noted that we obtain similar results when using the 43 proteins only that are in agreement with Suter et al. [38] or when using all 71 proteins.

An expansion of the polyQ tract of ATXN1 has been identified as the cause of the neurodegenerative disease SCA1 [1]. The overall function of wt polyQ stretch is likely to stabilize PPI and/or spacer elements between individual folded domains in molecules that mediate PPI [40, 59]. Evolutionary analyses, however, suggest that the polyQ tract does not seem to be necessary for protein function [40]. Indeed, neither the ATXN1L paralog, nor the ATXN1 orthologous genes of *M. musculus*, *X. tropicalis*, *X. laevis*, *D. rerio*, *D. melanogaster* and *C. elegans* show a polyQ tract. All these proteins present the AXH domain, and as here shown, all human proteins here studied interact at the C-terminal region (where the SAR and AXH domains are located), and not with the polyQ region. The AXH domain is essential for binding with ATXN1, in agreement with previous studies [9–13]. When the polyQ region is expanded, more interactions are established at the N-terminal region. This result is in agreement with the observation that a larger number of interactions are established with the N-terminal region of the expanded than with the wt ATXN1 [36]. Although we did not find evidence supporting the importance of the polyQ surrounding sequences on the PPI modulation [52], this may be due to difficulties in determining the structure of polyQ regions [31, 60, 61]. In the literature, the polyQ tract of the two ATXN1 forms has been proposed as being largely the same [62], slightly different in the overall secondary structure content [63], or even as a sharp change from an extended monomeric conformation to a collapsed state [64]. Here we used the models of both forms of ATXN1 with higher confidence scoring and with the most similar secondary structure.

For the N-terminal ATXN1 region, we identify motifs related with transcription, cell communication, phosphorylation, and sumoylation. The latter two are the major posttranslational modifications described for ATXN1 (see Background). 80% of the interactors that bind with expanded ATXN1 interact in these motif regions, thus showing the importance of these processes in SCA1. Moreover, it is conceivable that there is also a RNA binding domain in this region, and that the proteins that interact with expanded ATXN1 at the N-terminal region block the access of other proteins or RNA. Phosphorylation of serine residue 776 by Protein Kinase A (PKA) has been reported, but there are three other putative residues that could be phosphorylated (a Threonine at position 236 and a Serine at residues 239 and 254). The 776 residue is part of a canonical Arg-containing phospho-motif responsible for a strong interaction with 14–3-3 proteins, such as YWHAE. In mouse ATXN1, seven phosphorylation sites have been identified, and two are in the N-terminal region of the protein [65]. Kang and Hong [66] showed that the SUMO-1 protein interacts with mutant ATXN1, but not with wt ATXN1, suggesting the involvement of the SUMO-1 system in the pathogenesis of SCA1 disease.

## Conclusions

In conclusion, the *in-silico* method here used can provide useful insight into which interactors bind preferably to the expanded ATXN1, predict binding surfaces, and possible functional consequences of the expansion of the polyQ tract. Our results are 84.5% in agreement with the largest data set available using as baits ATXN1 full-length proteins [38]. The major limitations of the *in-silico* methodology are the size of the proteins considered (the prediction programs do not infer the structure of proteins larger than 1500 amino acids), as well as considering binary interactions only and not larger protein complexes. Such methodology can be used to address differences in the binding surfaces caused by the expansion of the polyQ in other diseases. Using this approach, we revealed the importance of the N-terminal region, before the expanded polyQ region of the ATXN1, where an increase in the number of interactions is predicted. In this region we identify motifs related with transcription, cell communication, phosphorylation, sumoylation, and RNA binding. In-vitro and in-vivo studies using mutations at these motifs are required to understand how they influence the function and regulation of the expanded ATXN1. A similar approach can be used to gain insight into the molecular basis of other neurodegenerative diseases.

## Supplementary information

**Additional file 1: Table S1.** The 311 proteins reported to interact with human ATXN1 according to EvoPPI, as well as those interactions reported in Suter et al. [38], Lim et al. [29] and Hosp et al. [37] in common with EvoPPI. Black squares represent presence of the ATXN1 interaction. Cells marked in blue report preferential interactions with wt ATXN1, in red preferential interactions with expanded ATXN1, in green interactions with both forms of ATXN1, and in grey those for which data regarding binding preference is not available.

**Additional file 2: Table S2.** The involvement of the 65 human proteins with polyQ in disease.

**Additional file 3: Figure S1.** Linear regression and 95% confidence interval for the number of PPI and length for the 65 *H. sapiens* polyQ proteins.

**Additional file 4: Table S3.** Proteins reported to interact with human ATXN1L. The cells marked in black represent common presence in ATXN1L and ATXN1. In bold are the proteins that preferentially bind to expanded ATXN1.

**Additional file 5: Table S4.** Number of interactors in agreement with wt and expanded ATXN1 using different methodologies. Prediction agreement for the number of interactors with wt and expanded ATXN1 when using the in-silico and Suter et al. [38] methodologies. The results are presented according to the number of residues in the interface (NRI) of the interacting partners, percentage of number of interface residues, solvent-accessible area (SAA), and percentage of the solvent-accessible area of wild-type ATXN1, expanded ATXN1 and interacting partners. In brackets are the number of cases that show agreement versus the total number of interactors analyzed.

**Additional file 6: Figure S2.** Residues homology of the main templates to predict the ATXN1 structure (I-TASSER [44]). The polyQ region is represented in red and the AXH domain in green.

**Additional file 7: Figure S3.** The interface residues of the crystal structures models of the AXH domain bound to CIC. The interface residues of the ATXN1 at the AXH domain in the crystal structures models of the AXH domain bound to CIC (PDB ID: 4J2L, [49] and PDB ID: 2 M41, [17]) are represented in red, and with the 43 interactors in agreement with Suter et al. [38] are represented in blue.

**Additional file 8: Figure S4.** Comparison of the docking results of the wt and expanded ATXN1 with the 71 proteins. The results are presented according to the: **A)** interfacing residues of the ATXN1, B) percentage of interfacing residues of the ATXN1, C) number of interfacing residues for the 43 interactors, D) percentage of interfacing residues of the 43 interactors, E) interface of the solvent accessible area (Å) of the ATXN1, and F) the percentage of the interface of the solvent accessible area of the ATXN1.

**Additional file 9: Figure S5.** RNApred SVM values along the ATXN1 using a sliding window of 50 residues and an increment of 25. The light grey box indicates the location of the polyQ region while the dark grey box indicates the location of the RNA binding region described in the literature.

**Additional file 10: Table S5**. Uniprot and gene ID, gene name(s), and protein size for ATXN1 and ATXN1L in the species used.

**Additional file 11: Figure S6.** Flowchart showing methodology 2 (the one showing the best agreement with Suter et al. [38])

**Additional file 12:.** List of PDB files describing the interaction of wt (ATXN1Q29) and expanded (ATXN1Q82) ATXN1 with 71 interactors. Files can be downloaded from Zenodo DOI: https://doi.org/10.5281/zenodo.3416591.

## Data Availability

*H. sapiens*, *M. musculus*, *X. laevis*, *D. rerio, D. melanogaster,* and *C. elegans* ATXN1 and ATXN1L PPI have been retrieved from 12 databases (BioGRID, CCSB, DroID, FlyBase, HIPPIE, HitPredict, HomoMINT, INstruct, Interactome3d, mentha, MINT, and/or PINA) using EvoPPI (accession numbers are listed in Additional file 10: Table S5). The 81 proteins, studied in Suter et al. [38], and their corresponding UniProtKB (https://www.uniprot.org/) accession numbers are: ATXN1 - P54253, ADD3 - Q9UEY8, ARID5A - Q03989, ASNS - P08243, BAALC - Q8WXS3, BASP1 - P80723, C16orf5 - Q9H305, C2orf27B - Q580R0, CAMK2B - Q13554, CHRNA7 - P36544, CLCN2 - P51788, CREM - Q03060, CRIP2 - P52943, CRK - P46108, CRY2 - Q49AN0, CXorf27 - O75409, DHRSX - Q8N5I4, DHX37 - Q8IY37, DIXDC1 - Q155Q3, DOCK5 - Q9H7D0, EHMT1 - Q9H9B1, EIF1B - O60739, EIF3F - O00303, ESRRA - P11474, ETV4 - P43268, FAM46A - Q96IP4, FAM46B - Q96A09, FAR1 - Q8WVX9, FOSL1 - P15407, FUBP3 - Q96I24, GATAD1 - Q8WUU5, GGA2 - Q9UJY4, HEY2 - Q9UBP5, HEYL - Q9NQ87, HNRPLL - Q8WVV9, IGF2R - P11717, IMMT - Q16891, ILVBL - A1L0T0, KCTD15 - Q96SI1, KIF22 - Q14807, LASP1 - Q14847, LITAF - Q99732, LOXL1 - Q08397, LPAR2 - Q9HBW0, MAGEB18 - Q96M61, MAGEB2 - O15479, MAGEB6 - Q8N7X4, MCART1 - Q9H1U9, MLST8 - Q9BVC4, MSX2 - P35548, NCAM1 - P13591, NPHP3 - Q7Z494, OTX2 - P32243, PIAS1 - O75925, PLEKHB1 - Q9UF11, PPAT - Q06203, PSPH - P78330, QKI - Q96PU8, RAI2 - Q9Y5P3, RAPGEF1 - Q13905, RBM26 - Q5T8P6, RBM9 - O43251, SEMA4G - Q9NTN9, SF1 - Q15637, SLC4A2 - P04920, SLC6A13 - Q9NSD5, STAM2 - O75886, SV2A - Q7L0J3, TCTA - P57738, TMX2 - Q9Y320, TOMM20 - Q15388, TP53I11 - O14683, TRIM38 - O00635, TSC1 - Q92574, TTRAP - O95551, UHRF1BP1L - A0JNW5, UHRF2 - Q96PU4, WBSCR16 - Q96I51, YWHAE - P62258, ZC3H10 - Q96K80, ZSCAN1 - Q8NBB4, ZXDC - Q2QGD7. The list of PDB files describing the docking of the proteins used in this study can be found in Additional file 12.

## Abbreviations

29Q: 29 polyglutamine
82Q: 82 polyglutamine
ATXN1: Ataxin-1
ATXN1L: Ataxin-1-like
Blast: Basic Local Alignment Search Tool
CIC: Capicua
GST: Glutathione-S-transferase
NLS: Nuclear localization signal
PDB: Protein data bank
PKA: Protein Kinase A
polyQ: polyglutamine
PPI: Protein-protein interactions
SAR: Self-association region
SCA1: Spinocerebellar ataxia type 1
wt: wild-type
Y2H: yeast two-hybrid
