# Supplementary material for: ATXN1 N-terminal region explains the binding differences of wild-type and expanded forms
Source: BMC Med Genomics. 2019 Oct 26;12:145. doi: 10.1186/s12920-019-0594-4 (PMC6814966; doi:10.1186/s12920-019-0594-4)
Supplement: Supplementary file 1 — Additional file 1: Table S1. The 311 proteins reported to interact with human ATXN1 according to EvoPPI, as well as those interactions reported in Suter et al. [38], Lim et al. [29] and Hosp et al. [37] in common with EvoPPI. Black squares represent presence of the ATXN1 interaction. Cells marked in blue report preferential interactions with wt ATXN1, in red preferential interactions with expanded ATXN1, in green interactions with both forms of ATXN1, and in grey those for which data regarding binding preference is not available. [file 12920_2019_594_MOESM1_ESM.pdf]

Additional file 1: Table S1. The 311 proteins reported to interact with human ATXN1 according to EvoPPI, as well as those interactions reported in Suter et al. [38], Lim et al. [36] and Hosp et al. [37] in common with EvoPPI. Black squares represent presence of the ATXN1 interaction. Cells marked in blue report preferential interactions with wt ATXN1, in red preferential interactions with expanded ATXN1, in green interactions with both forms of ATXN1, and in grey those for which data regarding binding preference is not available.

GeneID	Gene Name	mentha	HitPredict	HIPPIE	Pina2	BioGRID	HomoMINT	CCSB Lit_BM_13	CCSB HI_II_14	Mint	Instruct	Interactome3D	Suter et al. [38]	Lim et al. [36]	Hosp et al. [37]
31	ACACA	■	■	■	■	■								blue	
112	ADCY6	■	■	■	■	■	■							grey	
120	ADD3	■	■	■	■	■							blue		
207	AKT1			■	■										
322	APBB1	■	■	■	■	■	■							green	
440	ASNS	■	■	■	■	■							blue		
478	ATP1A3	■	■	■	■	■	■							green	
639	PRDM1	■	■	■	■	■	■							red	
816	CAMK2B	■	■	■	■	■							blue		
955	ENTPD6	■	■	■	■	■								red	
1021	CDK6	■	■	■	■	■	■							grey	
1072	CFL1	■	■	■	■	■	■	■						green	
1139	CHRNA7			■		■							green		
1153	CIRBP	■	■	■	■	■	■							grey	
1181	CLCN2	■	■	■	■	■							red		
1390	CREM	■	■	■	■	■							red		
1397	CRIP2	■	■	■	■	■							red		
1398	CRK	■	■	■	■	■	■	■					red	grey	
1406	CRX	■	■												
1408	CRY2	■	■	■	■	■							red		
1471	CST3	■	■	■	■	■	■							red	
1760	DMPK	■	■	■	■	■	■							red	
2101	ESRRA	■	■	■	■	■							red		
2118	ETV4	■	■	■	■	■							blue		
2137	EXTL3	■	■												
2224	FDPS	■	■	■	■	■	■							blue	
2274	FHL2	■	■	■	■	■	■							red	
2534	FYN	■	■	■	■	■	■							green	

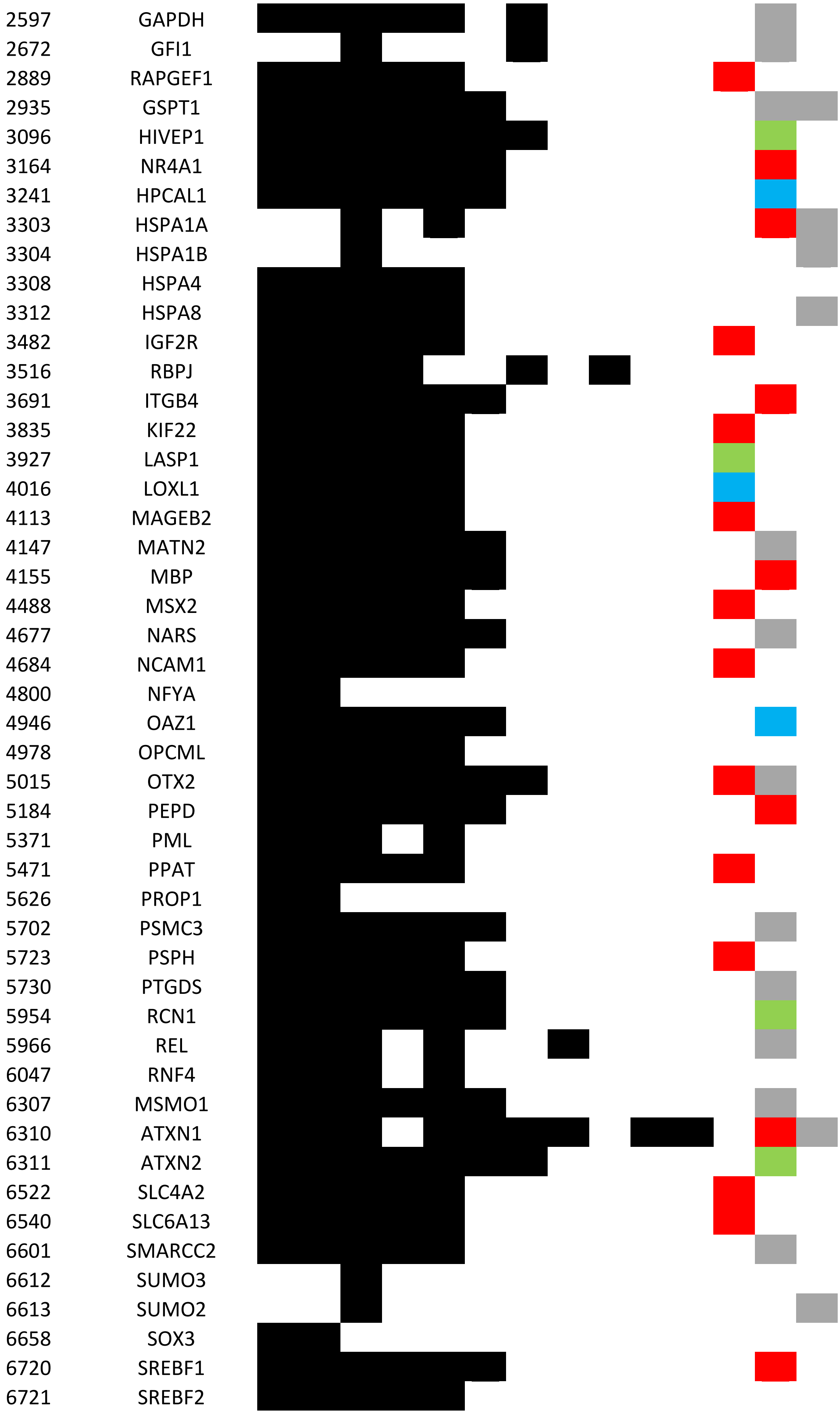

2597 GAPDH
2672 GFI1
2889 RAPGEF1
2935 GSPT1
3096 HIVEP1
3164 NR4A1
3241 HPCAL1
3303 HSPA1A
3304 HSPA1B
3308 HSPA4
3312 HSPA8
3482 IGF2R
3516 RBPJ
3691 ITGB4
3835 KIF22
3927 LASP1
4016 LOXL1
4113 MAGEB2
4147 MATN2
4155 MBP
4488 MSX2
4677 NARS
4684 NCAM1
4800 NFYA
4946 OAZ1
4978 OPCML
5015 OTX2
5184 PEPD
5371 PML
5471 PPAT
5626 PROP1
5702 PSMC3
5723 PSPH
5730 PTGDS
5954 RCN1
5966 REL
6047 RNF4
6307 MSMO1
6310 ATXN1
6311 ATXN2
6522 SLC4A2
6540 SLC6A13
6601 SMARCC2
6612 SUMO3
6613 SUMO2
6658 SOX3
6720 SREBF1
6721 SREBF2

6913	TBX15
6936	GCFC2
6988	TCTA
7186	TRAF2
7205	TRIP6
7248	TSC1
7316	UBC
7324	UBE2E1
7329	UBE2I
7341	SUMO1
7415	VCP
7447	VSNL1
7531	YWHAE
7534	YWHAZ
7536	SF1
7791	ZYX
7874	USP7
7916	PRRC2A
7994	KAT6A
8061	FOSL1
8125	ANP32A
8161	COIL
8301	PICALM
8350	HIST1H3A
8351	HIST1H3D
8352	HIST1H3C
8353	HIST1H3E
8354	HIST1H3I
8355	HIST1H3G
8356	HIST1H3J
8357	HIST1H3H
8358	HIST1H3B
8399	PLA2G10
8462	KLF11
8514	KCNAB2
8536	CAMK1
8553	BHLHE40
8554	PIAS1
8557	TCAP
8665	EIF3F
8751	ADAM15
8812	CCNK
8861	LDB1
8939	FUBP3
8968	HIST1H3F
9086	EIF1AY
9093	DNAJA3
9114	ATP6V0D1

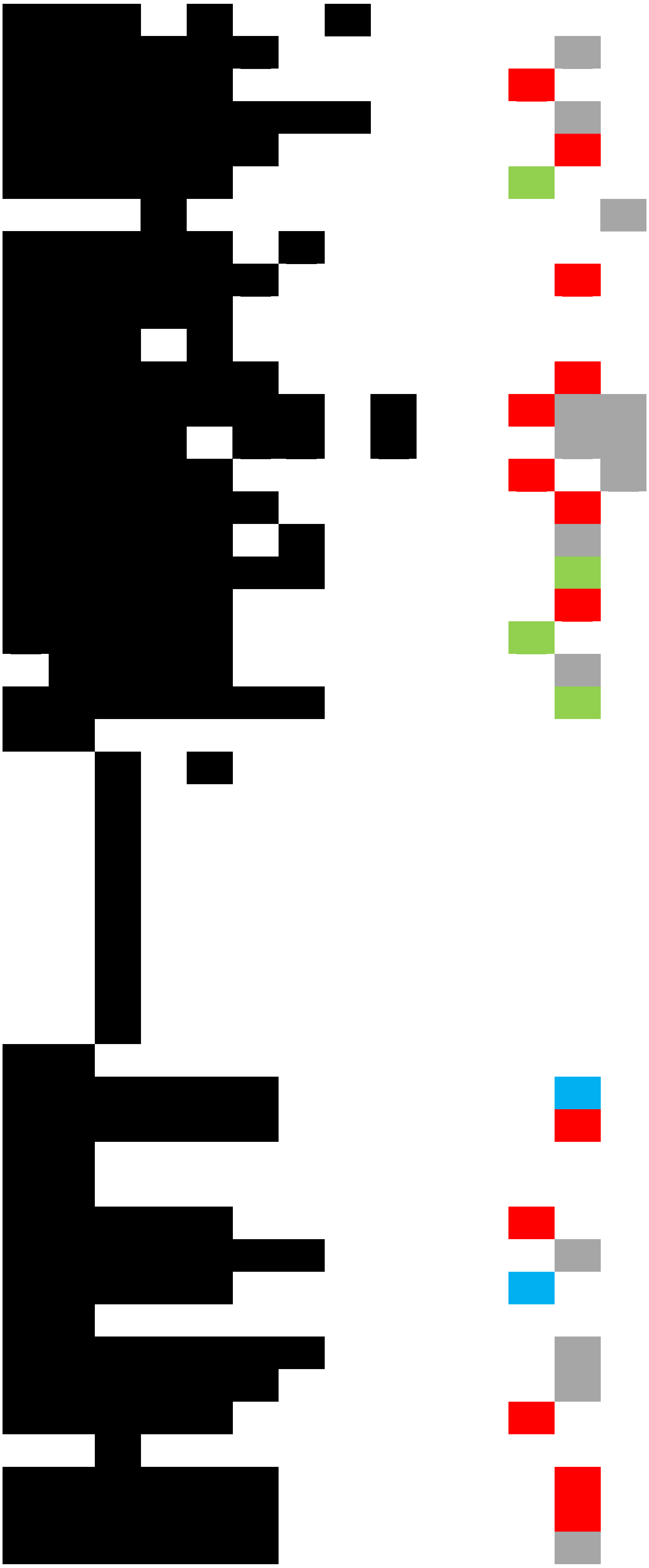

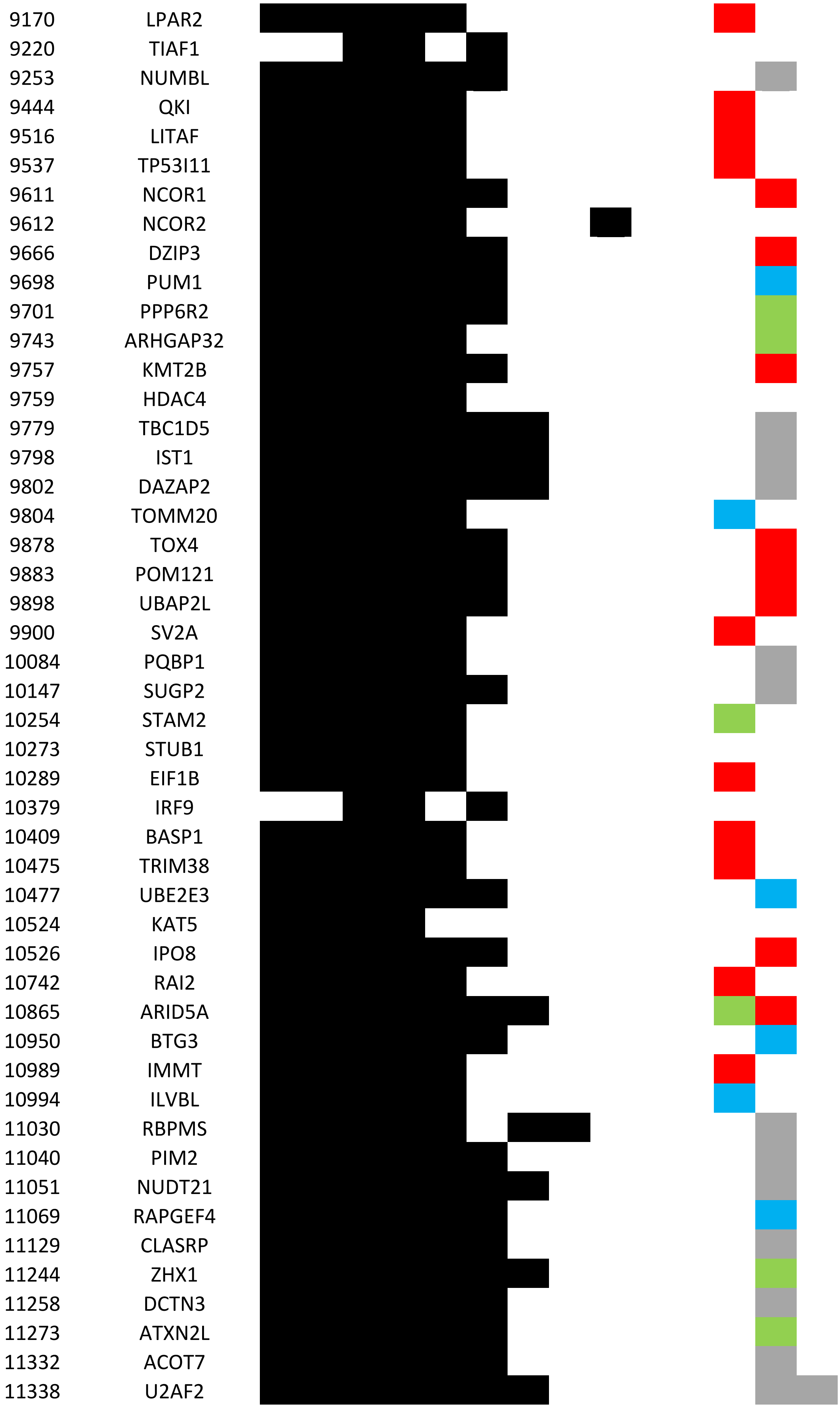
9170 LPAR2
9220 TIAF1
9253 NUMBL
9444 QKI
9516 LITAF
9537 TP53I11
9611 NCOR1
9612 NCOR2
9666 DZIP3
9698 PUM1
9701 PPP6R2
9743 ARHGAP32
9757 KMT2B
9759 HDAC4
9779 TBC1D5
9798 IST1
9802 DAZAP2
9804 TOMM20
9878 TOX4
9883 POM121
9898 UBAP2L
9900 SV2A
10084 PQBP1
10147 SUGP2
10254 STAM2
10273 STUB1
10289 EIF1B
10379 IRF9
10409 BASP1
10475 TRIM38
10477 UBE2E3
10524 KAT5
10526 IPO8
10742 RAI2
10865 ARID5A
10950 BTG3
10989 IMMT
10994 ILVBL
11030 RBPMS
11040 PIM2
11051 NUDT21
11069 RAPGEF4
11129 CLASRP
11244 ZHX1
11258 DCTN3
11273 ATXN2L
11332 ACOT7
11338 U2AF2

22809	ATF5
22864	R3HDM2
22882	ZHX2
22905	EPN2
22954	TRIM32
22986	SORCS3
23001	WDFY3
23013	SPEN
23051	ZHX3
23053	ZSWIM8
23060	ZNF609
23062	GGA2
23074	UHRF1BP1L
23126	POGZ
23131	GPATCH8
23132	RAD54L2
23152	CIC
23201	FAM168A
23264	ZC3H7B
23493	HEY2
23506	GLTSCR1L
23518	R3HDM1
23543	RBFOX2
23587	ELP5
25758	KIAA1549L
25763	HYPM
25801	GCA
26205	GMEB2
26508	HEYL
27031	NPHP3
27033	ZBTB32
27245	AHDC1
27304	MOCS3
29072	SETD2
29085	PHPT1
29798	C2orf27A
29965	CDIP1
30008	EFEMP2
30815	ST6GALNAC6
50804	MYEF2
51075	TMX2
51076	CUTC
51371	POMP
51567	TDP2
51586	MED15
51701	NLK
54472	TOLLIP
54477	PLEKHA5

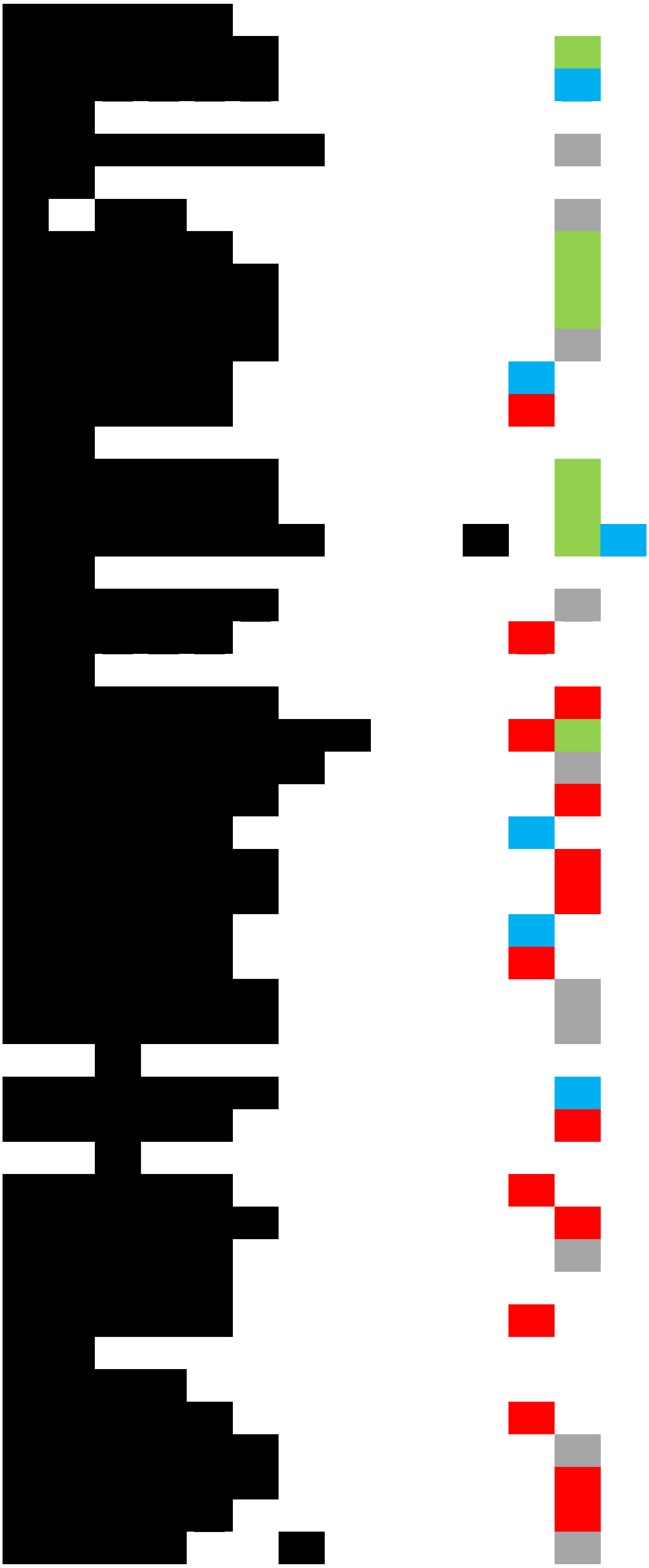

54540	FAM193B
54715	RBFOX1
54845	ESRP1
54870	QRICH1
54882	ANKHD1
54902	TTC19
55072	RNF31
55249	YY1AP1
55578	SUPT20H
55603	FAM46A
55638	SYBU
56478	EIF4ENIF1
56681	SAR1A
56893	UBQLN4
57055	DAZ2
57563	KLHL8
57647	DHX37
57715	SEMA4G
57798	GATAD1
58473	PLEKHB1
59349	KLHL12
64062	RBM26
64223	MLST8
64745	METTL17
64753	CCDC136
65125	WNK1
65268	WNK2
79047	KCTD15
79364	ZXDC
79633	FAT4
79813	EHMT1
79869	CPSF7
79870	BAALC
79918	SETD6
80005	DOCK5
81554	RCC1L
84069	PLEKHN1
84188	FAR1
84528	RHOXF2
84726	PRRC2B
84872	ZC3H10
84970	C1orf94
84991	RBM17
85451	UNK
85458	DIXDC1
90678	LRSAM1
91752	ZNF804A
92014	SLC25A51

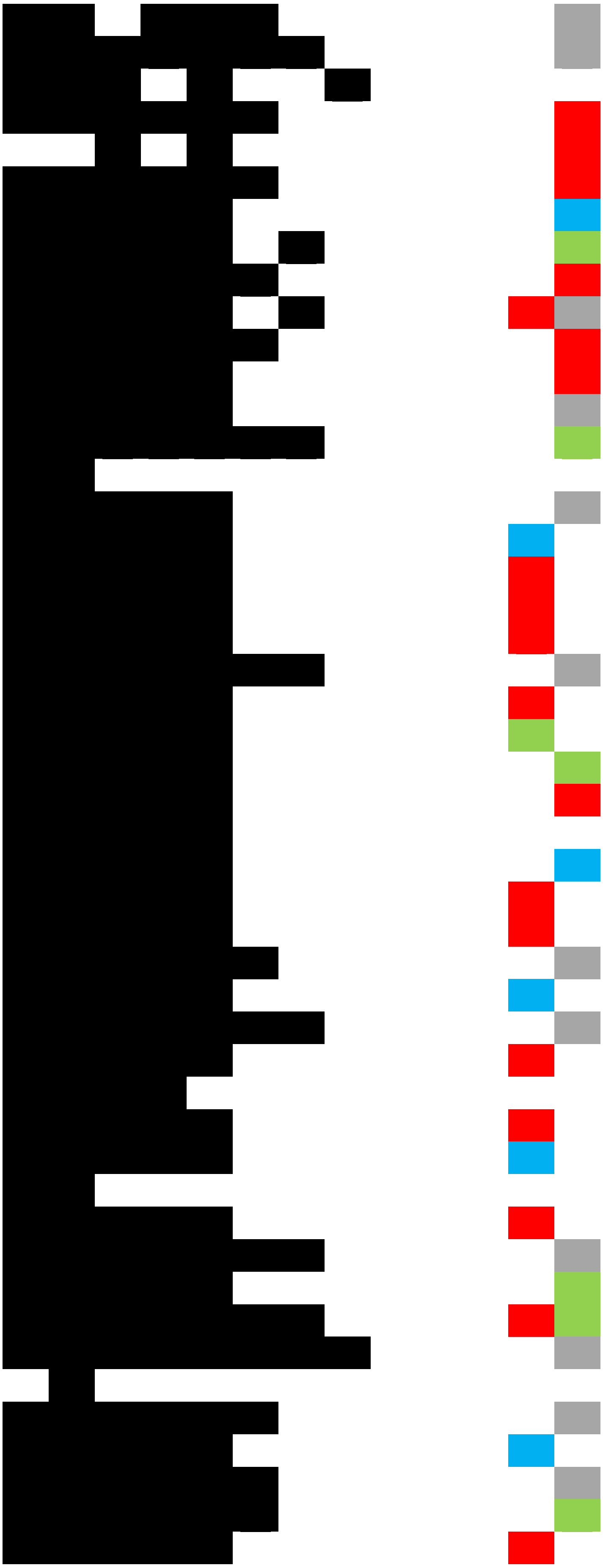

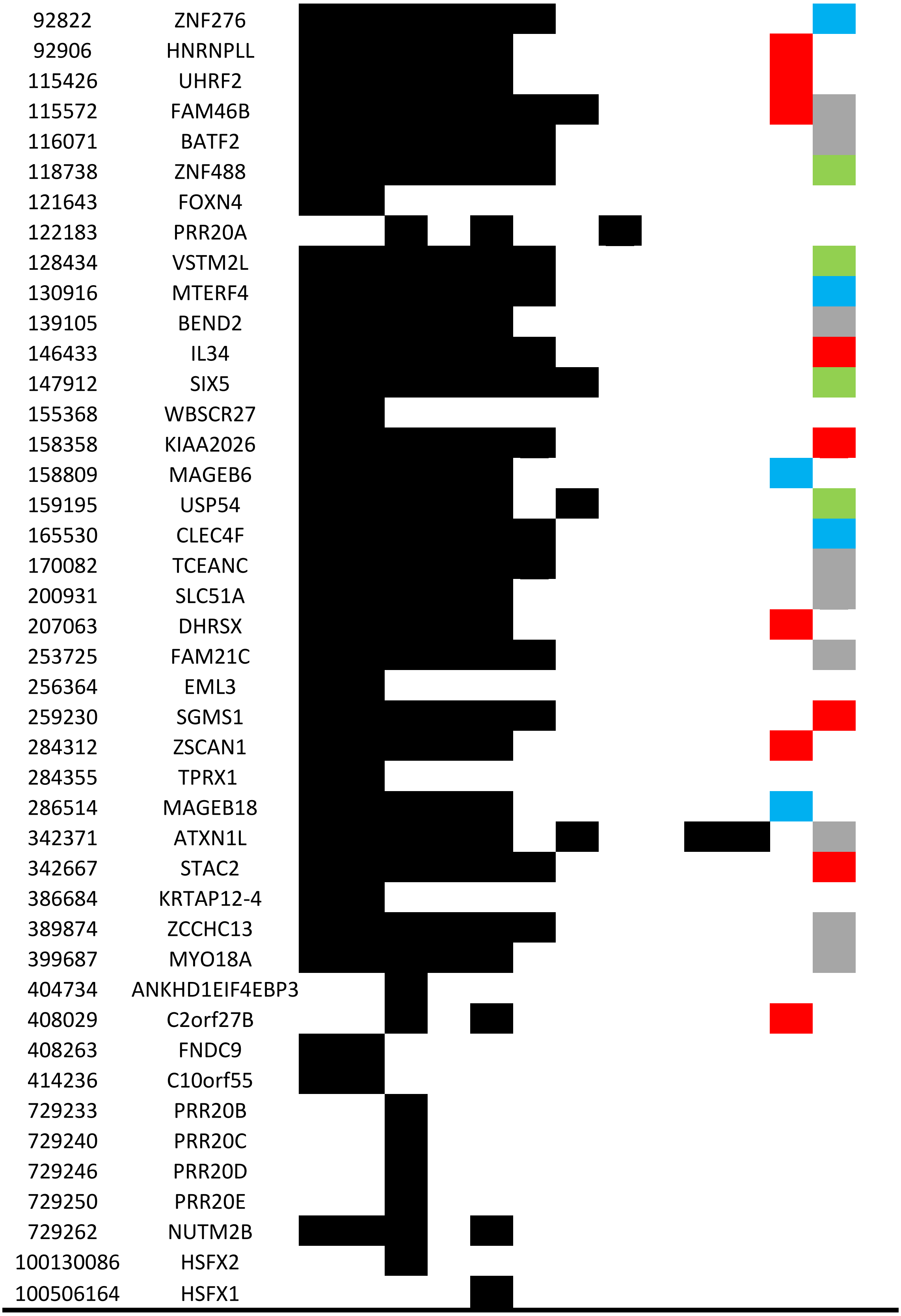
92822 ZNF276
92906 HNRNPLL
115426 UHRF2
115572 FAM46B
116071 BATF2
118738 ZNF488
121643 FOXN4
122183 PRR20A
128434 VSTM2L
130916 MTERF4
139105 BEND2
146433 IL34
147912 SIX5
155368 WBSCR27
158358 KIAA2026
158809 MAGEB6
159195 USP54
165530 CLEC4F
170082 TCEANC
200931 SLC51A
207063 DHRSX
253725 FAM21C
256364 EML3
259230 SGMS1
284312 ZSCAN1
284355 TPRX1
286514 MAGEB18
342371 ATXN1L
342667 STAC2
386684 KRTAP12-4
389874 ZCCHC13
399687 MYO18A
404734 ANKHD1EIF4EBP3
408029 C2orf27B
408263 FNDC9
414236 C10orf55
729233 PRR20B
729240 PRR20C
729246 PRR20D
729250 PRR20E
729262 NUTM2B
100130086 HSFX2
100506164 HSFX1
