# Supplementary material for: ATXN1 N-terminal region explains the binding differences of wild-type and expanded forms
Source: BMC Med Genomics. 2019 Oct 26;12:145. doi: 10.1186/s12920-019-0594-4 (PMC6814966; doi:10.1186/s12920-019-0594-4)
Supplement: Supplementary file 2 — Additional file 2: Table S2. The involvement of the 65 human proteins with polyQ in disease. [file 12920_2019_594_MOESM2_ESM.pdf]

Additional file 2: Table S2. The involvement of the 65 human proteins with polyQ in disease

Gene ID	Protein name	UniProtKB	Involvement in disease (according to NCBI- Gene;UniProt;and OMIM)
6310	ATXN1, ATX1, SCA1	P54253	Spinocerebellar ataxia 1 (SCA1)
367	AR, DHTR, NR3C4	P10275	Testicular feminization syndrome (TFM), and in Kennedy spinal and bulbar muscular atrophy (SBMA)
1822	ATN1, D12S755E, DRPLA	P54259	Dentatorubro-pallidoluysian atrophy (DRPLA)
6311	ATXN2, ATX2, SCA2, TNRC13	Q99700	Spinocerebellar ataxia 2 (SCA2); Amyotrophic lateral sclerosis 13 (ALS13); Parkinson disease, late-onset, susceptibility to
4287	ATXN3, ATX3, MJD, MJD1, SCA3	P54252	Machado-Joseph disease (MJD)
6314	ATXN7, SCA7	O15265	Spinocerebellar ataxia 7 (SCA7)
773	CACNA1A, CACH4, CACN3, CACNL1A4	O00555	Epileptic encephalopathy, early infantile; Episodic ataxia, type 2; Migraine, familial hemiplegic, 1; Migraine, familial hemiplegic, 1, with progressive cerebellar ataxia; Spinocerebellar ataxia 6 (SCA6)
3064	HTT, HD, IT15	P42858	Huntington disease (HD); Lopes-Maciel-Rodan syndrome (LOMARS)
6908	TBP, GTF2D1, TF2D, TFIID	P20226	Spinocerebellar ataxia 17 (SCA17); susceptibility to Parkinson disease
22848	AAK1, KIAA1048	Q2M2I8	Not Described
23	ABCF1, ABC50	Q8NE71	Play a role in enhancement of protein synthesis and the inflammation process
57492	ARID1B, BAF250B, DAN15, KIAA1235, OSA2	Q8NFD5	Coffin-Siris syndrome 1
10620	ARID3B, BDP, DRIL2	Q8IVW6	Present in K-562 erythrocytic leukemia cell line
429	ASCL1, ASH1, BHLHA46, HASH1	P50553	Highly expressed in 2 neuroendocrine cancers, medullary thyroid cancer (MTC; 155240) and small cell lung cancer (SCLC). Central hypoventilation syndrome, congenital, Haddad syndrome.
55589	BMP2K, BIKE, HRIHFB2017	Q9NSY1	Not Described
11189	CELF3, BRUNOL1, CAGH4, ERDA4, TNRC4	Q5SZQ8	Not Described
10523	CHERP, DAN26, SCAF6	Q8IWX8	Not Described
1387	CREBBP, CBP	Q92793	Rubinstein-Taybi syndrome 1; acute myeloid leukemia

196513	DCP1B	Q8IZD4	Not Described
9909	DENND4B, KIAA0476	O75064	Not Described
1750	DLX6	P56179	Not Described
57634	EP400, CAGH32, KIAA1498, KIAA1818, TNRC12	Q96L91	Not Described
165215	FAM171B, KIAA1946, NPD019	Q6P995	Not Described
93986	FOXP2, CAGH44, TNRC10	O15409	Speech-language disorder-1
84443	FRMPD3, KIAA1817	Q5JV73	Not Described
2734	GLG1, CFR1, ESL1, MG160	Q92896	Not Described
64207	IRF2BPL, C14orf4, EAP1, KIAA1865, My03	Q9H1B7	Neurodevelopmental disorder with regression, abnormal movements, loss of speech, and seizures
3782	KCNN3, K3	Q9UGI6	Possible Association with Schizophrenia
8085	KMT2D, ALR, MLL2, MLL4	KMT2D	Kabuki syndrome 1
8825	LIN7A, MALS1, VELI1	O14910	LIN7A is a major determinant of cell-polarity defects in breast carcinomas
9223	MAGI1, AIP3, BAIAP1, BAP1, TNRC19	Q96QZ7	Not Described
84441	MAML2, KIAA1819	Q8IZL2	Mucoepidermoid salivary gland carcinoma
55534	MAML3, KIAA1816	Q96JK9	Not Described
10046	MAMLD1, CG1, CXorf6	Q13495	Hypospadias 2, X-linked
9968	MED12, ARC240, CAGH45, HOPA, KIAA0192, TNRC11	Q93074	Lujan-Fryns syndrome; Ohdo syndrome, X-linked; Opitz-Kaveggia syndrome
51586	MED15, ARC105, CTG7A, PCQAP, TIG1, TNRC7	Q96RN5	DiGeorge syndrome
4205	MEF2A, MEF2	Q02078	Autosomal dominant coronary artery disease 1 with myocardial infarction (ADCAD1)
4330	MN1	Q10571	Meningioma
8202	NCOA3, AIB1, BHLHE42, RAC3, TRAM1	Q9Y6Q9	Breast and ovarian cancers

23054	NCOA6, AIB3, KIAA0181, RAP250, TRBP	Q14686	Colon, breast and lung cancers.
9612	NCOR2, CTG26	Q9Y618	Aberrant expression of this gene is associated with certain cancers. Alt
10725	NFAT5, KIAA0827, TONEBP	O94916	Invasive human ductal breast carcinomas.
9253	NUMBL	Q9Y6R0	Numblike regulates proliferation, apoptosis, and invasion of lung cancer cell.
1911	PHC1, EDR1, PH1	P78364	Microcephaly 11, primary, autosomal recessive (MCPH11)
22822	PHLDA1, PHRIP, TDAG51	Q8WV24	Myotonic dystrophy
5428	POLG, MDP1, POLG1, POLGA	P54098	Mitochondrial DNA depletion syndrome 4A (Alpers type); Mitochondrial DNA depletion syndrome 4B (MNGIE type); Mitochondrial recessive ataxia syndrome (includes SANDO and SCAE); Progressive external ophthalmoplegia, autosomal dominant 1; Progressive external ophthalmoplegia, autosomal recessive 1
5454	POU3F2, BRN2, OCT7, OTF7	P20265	Not Described
11281	POU6F2, RPF1	P78424	Wilms tumor susceptibility-5
22864	R3HDM2, KIAA1002	Q9Y2K5	Not Described
10743	RAI1, KIAA1820	Q7Z5J4	Smith-Magenis syndrome (SMS)
860	RUNX2, AML3, CBFA1, OSF2, PEBP2A	Q13950	Cleidocranial dysplasia; Cleidocranial dysplasia, forme fruste, dental anomalies only; Cleidocranial dysplasia, forme fruste, with brachydactyly; Metaphyseal dysplasia with maxillary hypoplasia with or without brachydactyly
6304	SATB1	Q01826	Delineates specific epigenetic modifications at target gene loci, directly up-regulating metastasis-associated genes while down-regulating tumor-suppressor genes. Reprograms chromatin organization and the transcription profiles of breast tumors to promote growth and metastasis.
23387	SIK3, KIAA0999, QSK, L19	Q9Y2K2	Somatic mutation in a breast cancer sample; expression elevated in ovarian cancers,
6595	SMARCA2, BAF190B, BRM, SNF2A, SNF2L2	P51531	Nicolaides-Baraitser syndrome
81849	ST6GALNAC5, SIAT7E	Q9BVH7	Not Described
7024	TFCP2, LSF, SEF	Q12800	May be involved in the pathogenesis of Alzheimer's disease
7942	TFEB, BHLHE35	P19484	Renal carcinoma
57215	THAP11, HRIHFB2206	Q96EK4	Not Described
730130	TMEM229A	B2RXF0	Not Described
27324	TOX3, CAGF9, TNRC9	O15405	A minor allele of this gene has been implicated in an elevated risk of breast cancer.

8848	TSC22D1, KIAA1994, TGFB1I4, TSC22, hucep-2	Q15714	May play a critical role in tumor suppression through the induction of cancer cell apoptosis, and a single nucleotide polymorphism in the promoter of this gene has been associated with diabetic nephropathy.
205717	USF3, KIAA2018	Q68DE3	A deletion in the polyglutamine region was associated with risk for thyroid carcinoma.
7716	VEZF1, DB1, ZNF161	Q14119	Involved in both normal and abnormal cellular proliferation and differentiation.
463	ZFHX3, ATBF1	Q15911	Prostate cancer, susceptibility to, somatic
171017	ZNF384, CAGH1, CIZ, NMP4, TNRC1	Q8TF68	Acute leukemia
