# Supplementary figures and images for: ATXN1 N-terminal region explains the binding differences of wild-type and expanded forms
Source: BMC Med Genomics. 2019 Oct 26;12:145. doi: 10.1186/s12920-019-0594-4 (PMC6814966; doi:10.1186/s12920-019-0594-4)

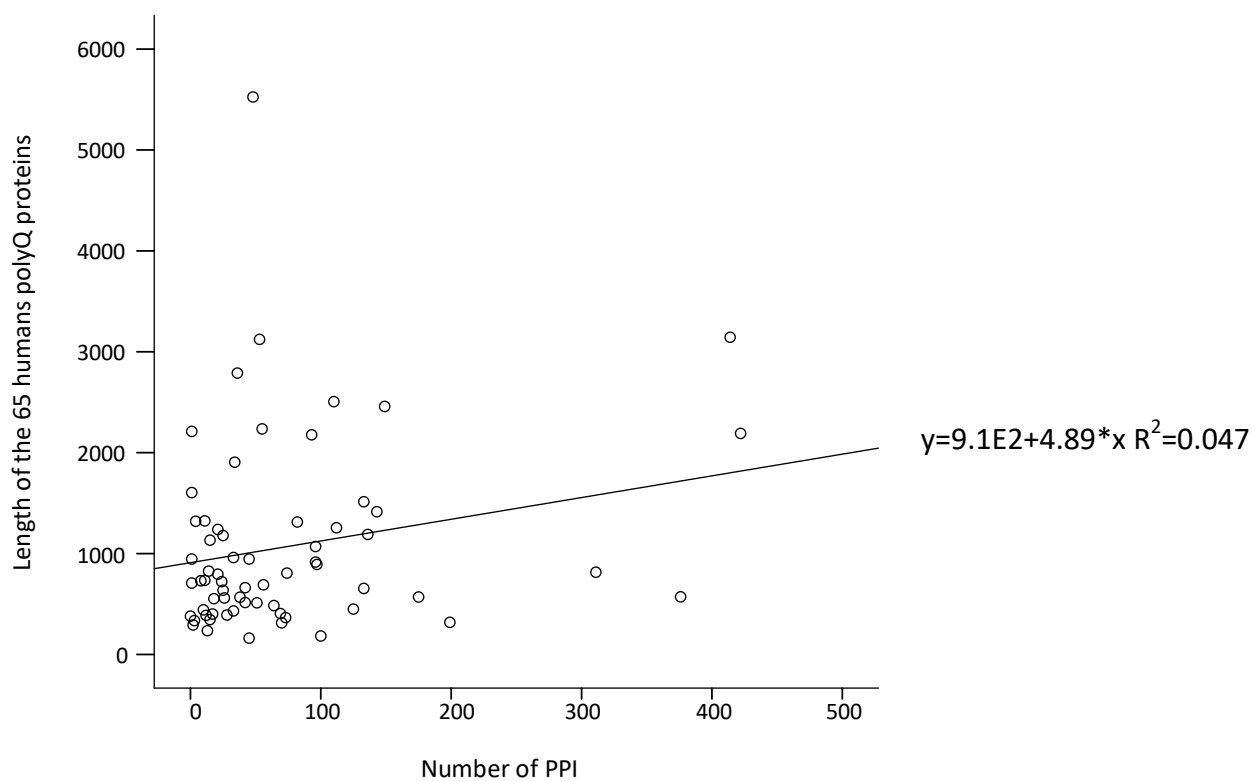

Additional file 3: Fig. S1

Supplement: Supplementary file 3 — Additional file 3: Figure S1. Linear regression and 95% confidence interval for the number of PPI and length for the 65 H. sapiens polyQ proteins. [file 12920_2019_594_MOESM3_ESM.pdf]

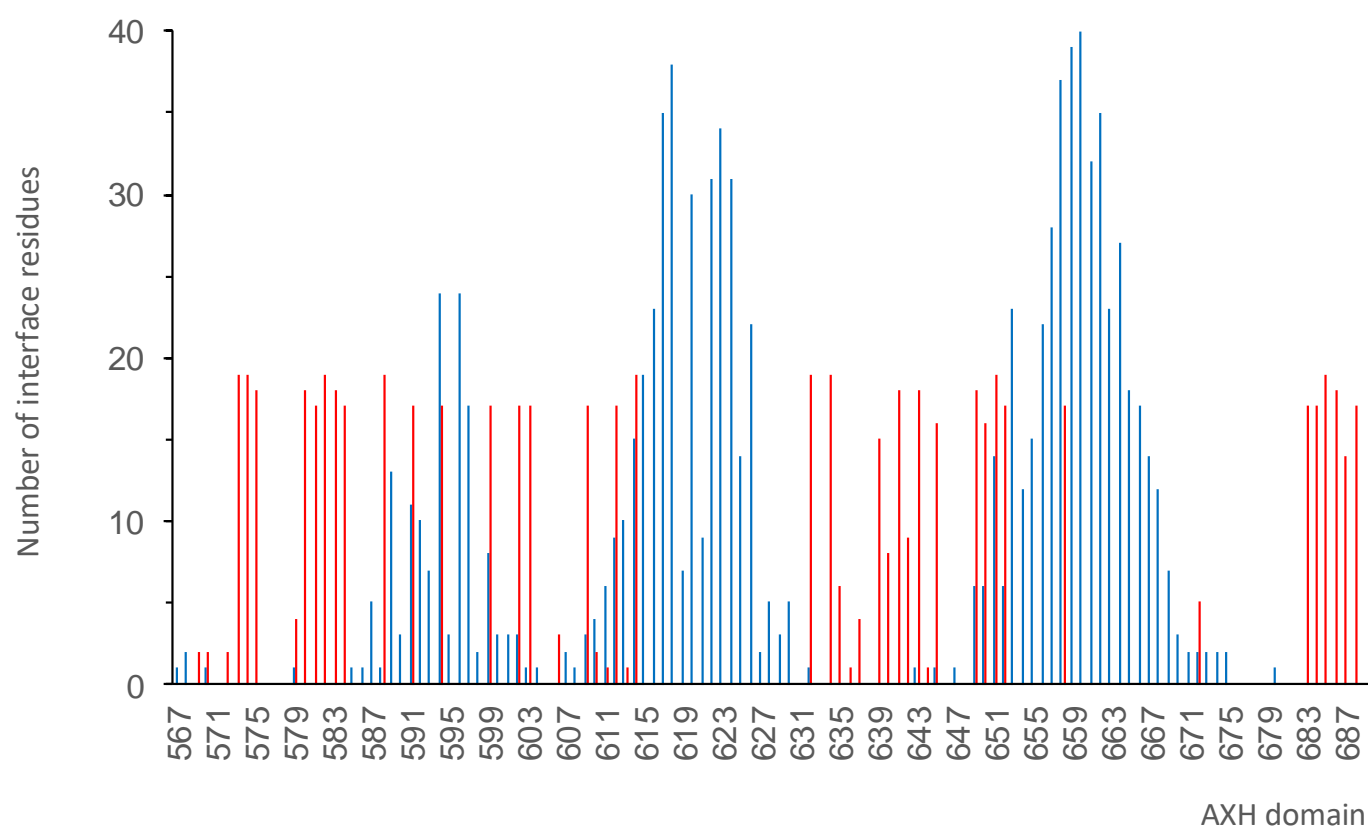

**Additional file 7: Fig. S3**

Supplement: Supplementary file 7 — Additional file 7: Figure S3. The interface residues of the crystal structures models of the AXH domain bound to CIC. The interface residues of the ATXN1 at the AXH domain in the crystal structures models of the AXH domain bound to CIC (PDB ID: 4J2L, [49] and PDB ID: 2 M41, [17]) are represented in red, and with the 43 interactors in agreement with Suter et al. [38] are represented in blue. [file 12920_2019_594_MOESM7_ESM.pdf]

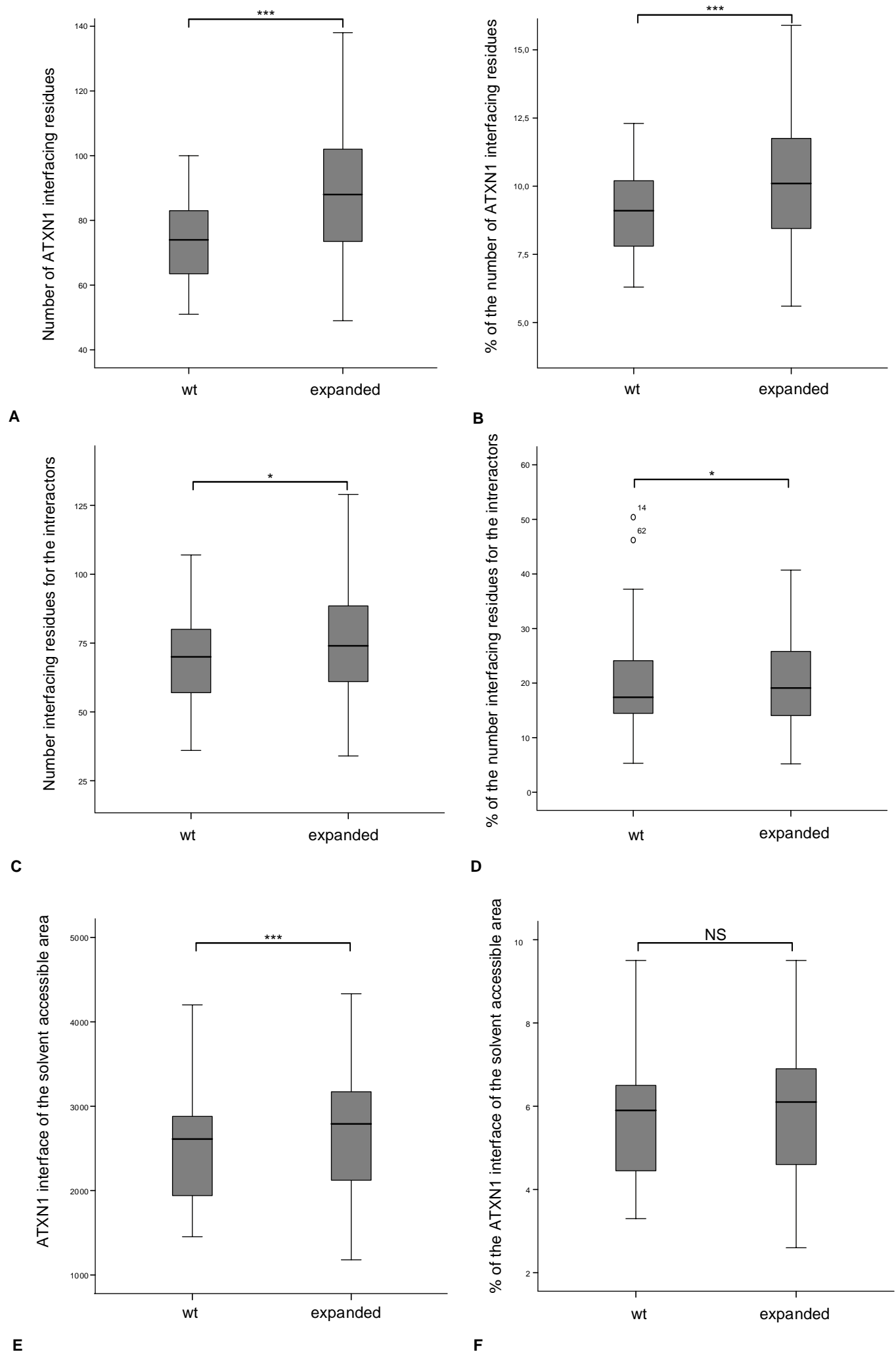

Additional file 8: Fig. S4

Supplement: Supplementary file 8 — Additional file 8: Figure S4. Comparison of the docking results of the wt and expanded ATXN1 with the 71 proteins. The results are presented according to the: A) interfacing residues of the ATXN1, B) percentage of interfacing residues of the ATXN1, C) number of interfacing residues for the 43 interactors, D) percentage of interfacing residues of the 43 interactors, E) interface of the solvent accessible area (Å) of the ATXN1, and F) the percentage of the interface of the solvent accessible area of the ATXN1. [file 12920_2019_594_MOESM8_ESM.pdf]

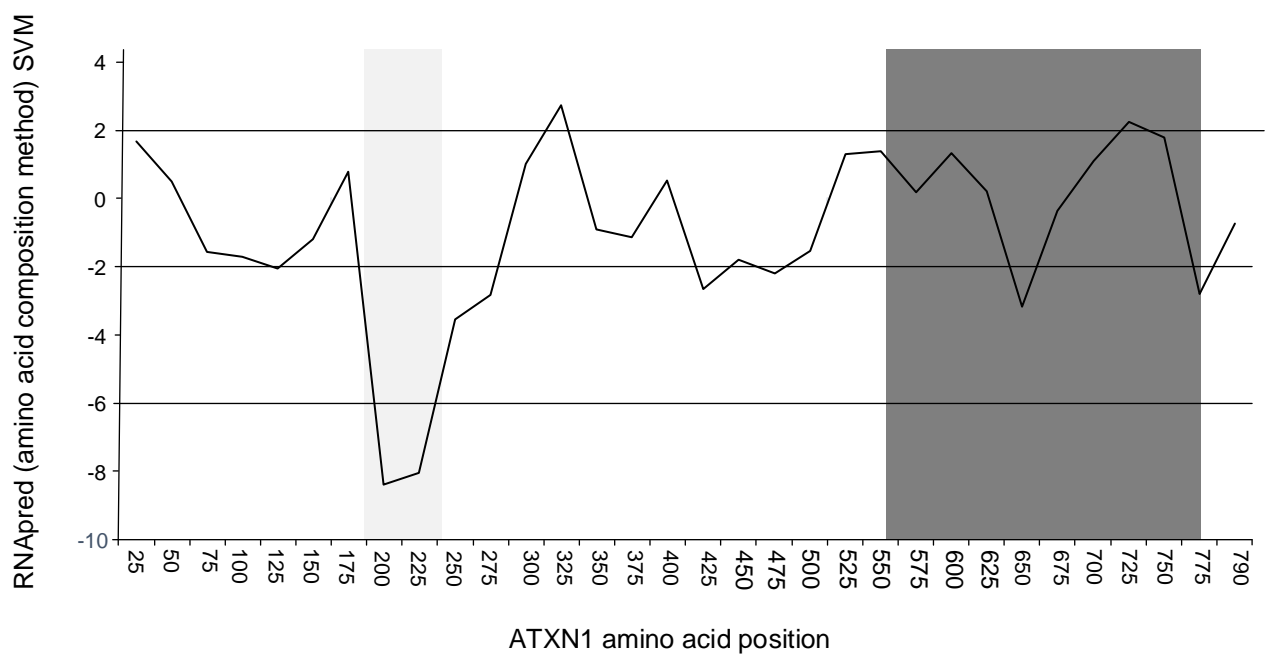

**Additional file 9: Fig. S5**

Supplement: Supplementary file 9 — Additional file 9: Figure S5. RNApred SVM values along the ATXN1 using a sliding window of 50 residues and an increment of 25. The light grey box indicates the location of the polyQ region while the dark grey box indicates the location of the RNA binding region described in the literature. [file 12920_2019_594_MOESM9_ESM.pdf]

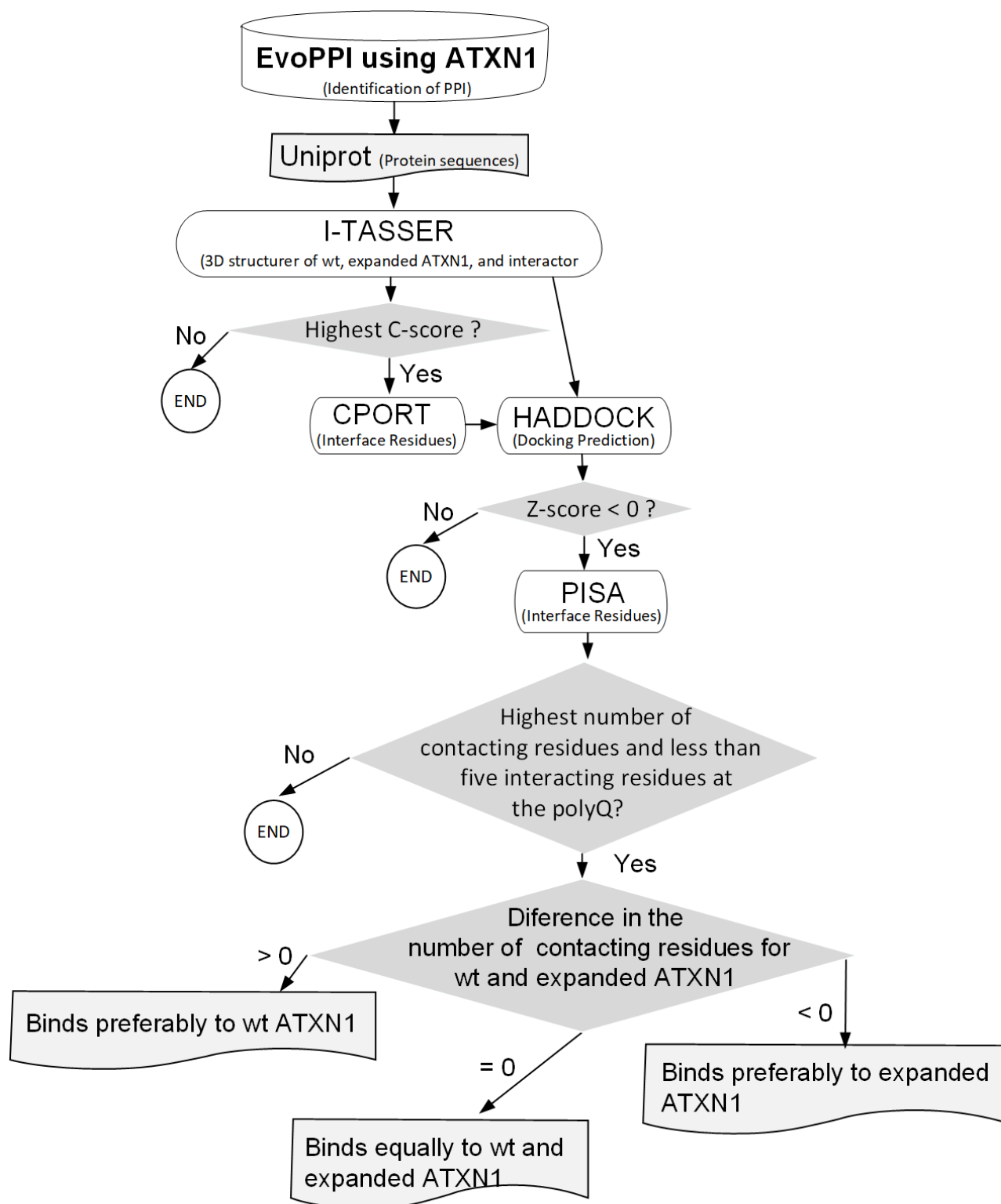

Additional file 11: Fig. S6

Supplement: Supplementary file 11 — Additional file 11: Figure S6. Flowchart showing methodology 2 (the one showing the best agreement with Suter et al. [38]) [file 12920_2019_594_MOESM11_ESM.pdf]
