# Supplementary material for: ATXN1 N-terminal region explains the binding differences of wild-type and expanded forms
Source: BMC Med Genomics. 2019 Oct 26;12:145. doi: 10.1186/s12920-019-0594-4 (PMC6814966; doi:10.1186/s12920-019-0594-4)
Supplement: Supplementary file 4 — Additional file 4: Table S3. Proteins reported to interact with human ATXN1L. The cells marked in black represent common presence in ATXN1L and ATXN1. In bold are the proteins that preferentially bind to expanded ATXN1. [file 12920_2019_594_MOESM4_ESM.pdf]

Additional file 4: Table S3. Proteins reported to interact with human ATXN1L. The cells marked in black represent common presence in ATXN1L and ATXN1. In bold are the proteins that preferentially bind to expanded ATXN1.

GeneID	Gene Name	In common with ATXN1
50	ACO2	
134	ADORA1	
166	AES	
322	APBB1	■
1173	AP2M1	
1487	CTBP1	
1488	CTBP2	
1601	DAB2	
2037	EPB41L2	
2521	FUS	
3096	HIVEP1	■
3516	RBPJ	■
4077	NBR1	
4147	MATN2	■
4152	MBD1	
4223	MEOX2	
4817	NIT1	
4914	NTRK1	
6310	ATXN1	■
6601	SMARCC2	■
6720	**SREBF1**	■
7070	THY1	
7916	PRRC2A	■
8161	COIL	■
8522	GAS7	
8906	AP1G2	
9139	CBFA2T2	
9146	HGS	
9169	SCAF11	
9321	TRIP11	
9415	FADS2	
9463	PICK1	
9555	H2AFY	
9611	**NCOR1**	■
9612	NCOR2	■
9802	DAZAP2	■
10147	SUGP2	■
10472	ZBTB18	
11030	RBPMS	■
11129	CLASRP	■
11332	ACOT7	■

23053	ZSWIM8	[redacted]
23131	GPATCH8	[redacted]
23132	RAD54L2	[redacted]
23136	EPB41L3	
23264	ZC3H7B	[redacted]
23271	CAMSAP2	
23543	**RBFOX2**	[redacted]
23587	ELP5	[redacted]
26003	GORASP2	
26205	**GMEB2**	[redacted]
29085	**PHPT1**	[redacted]
30815	ST6GALNAC6	[redacted]
51754	TMEM8B	
54882	**ANKHD1**	[redacted]
55249	YY1AP1	[redacted]
64121	RRAGC	
64745	METTL17	[redacted]
79868	ALG13	
84181	CHD6	
84632	AFAP1L2	
84726	PRRC2B	[redacted]
84866	TMEM25	
114785	MBD6	
159195	USP54	[redacted]
342371	ATXN1L	[redacted]
342667	**STAC2**	[redacted]
404734	ANKHD1EIF4EBP3	[redacted]
100130086	HSFX2	[redacted]
100506164	HSFX1	[redacted]
