# Supplementary material for: ATXN1 N-terminal region explains the binding differences of wild-type and expanded forms
Source: BMC Med Genomics. 2019 Oct 26;12:145. doi: 10.1186/s12920-019-0594-4 (PMC6814966; doi:10.1186/s12920-019-0594-4)
Supplement: Supplementary file 5 — Additional file 5: Table S4. Number of interactors in agreement with wt and expanded ATXN1 using different methodologies. Prediction agreement for the number of interactors with wt and expanded ATXN1 when using the in-silico and Suter et al. [38] methodologies. The results are presented according to the number of residues in the interface (NRI) of the interacting partners, percentage of number of interface residues, solvent-accessible area (SAA), and percentage of the solvent-accessible area of wild-type ATXN1, expanded ATXN1 and interacting partners. In brackets are the number of cases that show agreement versus the total number of interactors analyzed. [file 12920_2019_594_MOESM5_ESM.pdf]

Additional file 5: Table S4. Prediction agreement for the number of interactors with wt and expanded ATXN1 when using the in-silico and Suter et al. [38] methodologies

Parameters	Prediction agreement (%)	
NRI of the ATXN1	wt ATXN1	27.3 (3/11)
	expanded ATXN1	66.7 (40/60)
% NRI of the ATXN1	wt ATXN1	29.4 (5/17)
	expanded ATXN1	66.7 (36/54)
NRI of the interactor	wt ATXN1	28.0 (7/25)
	expanded ATXN1	71.7 (33/46)
% NRI of the interactor	wt ATXN1	28.0 (7/25)
	expanded ATXN1	71.7 (33/46)
SAA of the ATXN1	wt ATXN1	20.0 (4/20)
	expanded ATXN1	64.7 (33/51)
% SAA of the ATXN1	wt ATXN1	24.0 (6/25)
	expanded ATXN1	71.8 (28/39)
SAA of the interactor	wt ATXN1	41.2 (3/17)
	expanded ATXN1	64.8 (35/54)
% SAA of the interactor	wt ATXN1	18.8 (3/16)
	expanded ATXN1	64.2 (34/53)

Note: In brackets are the number of cases that show agreement versus the total number of interactors analyzed.
