# Supplementary material for: ATXN1 N-terminal region explains the binding differences of wild-type and expanded forms
Source: BMC Med Genomics. 2019 Oct 26;12:145. doi: 10.1186/s12920-019-0594-4 (PMC6814966; doi:10.1186/s12920-019-0594-4)
Supplement: Supplementary file 10 — Additional file 10: Table S5. Uniprot and gene ID, gene name(s), and protein size for ATXN1 and ATXN1L in the species used. [file 12920_2019_594_MOESM10_ESM.pdf]

Additional file 10: Table S5. Uniprot and gene ID, gene name(s), and protein size for ATXN1 and ATXN1L in the species used

Protein	Model Organism	UniProt	GeneID	Gene name(s)	Protein size (aa)
ATXN1					
	Homo sapiens	P54253	6310	*ATXN1, ATX1, SCA1*	815
	Mus musculus	P54254	20238	*Atxn1, Sca1*	791
	Xenopus tropicalis	F6PW06	100492711	*atxn1*	775
	Danio rerio (Ataxin 1a)	Q1L966	557340	*atxn1a, atxn1b*	781
	D. rerio (Ataxin 1b)	E7F6Z5	565841	*atxn1b*	827
	Drosophila melanogaster	Q9W3V7	31624	*Atx-1, CG454RA, dAtx-1, CG4547,*	230
	Caenorhabditis elegans	O44771	187002	*CELE_K04F10.1, K04F10.1*	299
ATXN1-like					
	H. sapiens	P0C7T5	342371	*ATXN1L, BOAT, BOAT1*	689
	M. musculus	P0C7T6	52335	*Atxn1l, Boat*	687
	X. laevis	B1WBC1	100158423	*atxn1l, LOC100158423, XELAEV_18024898mg*	691
	D .rerio	E7FB74	793927	*atxn1l*	765
