# Supplementary material for: ATXN1 N-terminal region explains the binding differences of wild-type and expanded forms
Source: BMC Med Genomics. 2019 Oct 26;12:145. doi: 10.1186/s12920-019-0594-4 (PMC6814966; doi:10.1186/s12920-019-0594-4)
Supplement: Supplementary file 6 — Additional file 6: Figure S2. Residues homology of the main templates to predict the ATXN1 structure (I-TASSER [44]). The polyQ region is represented in red and the AXH domain in green. [file 12920_2019_594_MOESM6_ESM.pdf]

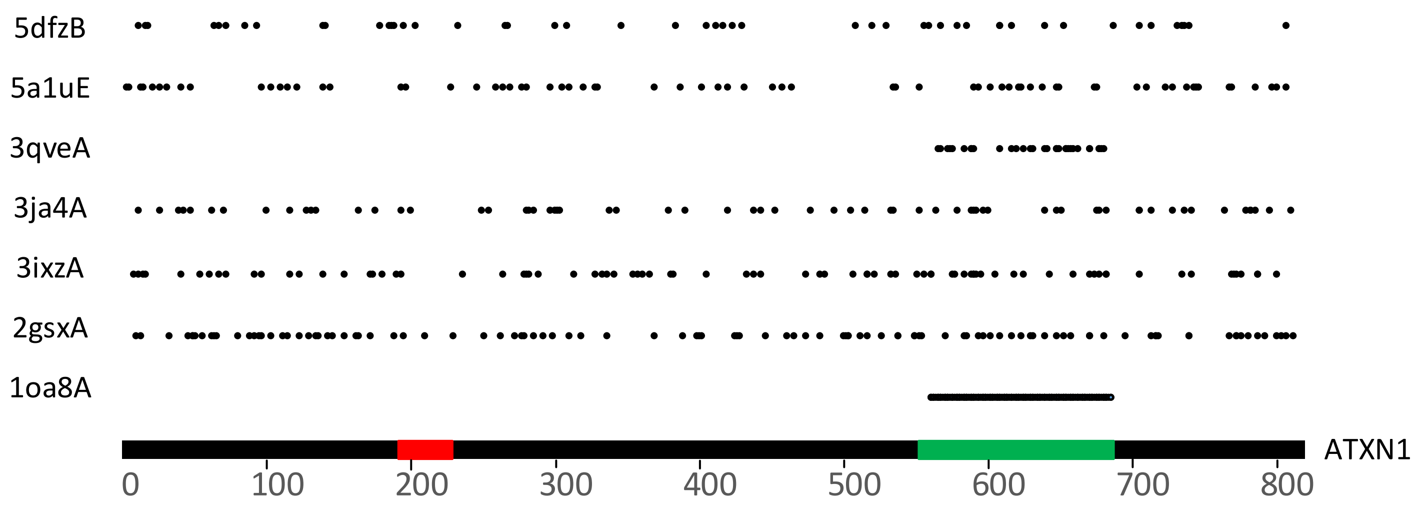

Additional file 6: Fig. S2
